# Efficient Approach to Carbinol Derivatives through Palladium-Catalyzed Base-Free Addition of Aryltriolborates to Aldehydes

**DOI:** 10.3390/molecules22091580

**Published:** 2017-09-20

**Authors:** Kun Hu, Pengqing Ye, Qianqian Zhen, Xinrong Yao, Tong Xu, Yinlin Shao, Jiuxi Chen

**Affiliations:** College of Chemistry & Materials Engineering, Wenzhou University, Wenzhou 325035, China; hukun15@163.com (K.H.); yepengqing17@163.com (P.Y.); wzuzqq0629@163.com (Q.Z.); yaoxinrong17@163.com (X.Y.); xuchentong17@163.com (T.X.); jiuxichen@wzu.edu.cn (J.C.)

**Keywords:** palladium-catalyzed, base-free, carbinol derivatives, aryltriolborates, aldehydes

## Abstract

Palladium-catalyzed base-free addition of aryltriolborates to aldehydes has been developed, leading to a wide range of carbinol derivatives in good to excellent yields. The efficiency of this transformation was demonstrated by compatibility with a wide range of functional groups. The present synthetic route to carbinol derivatives could be readily scaled up to gram quantity without difficulty. Thus, this method represents a simple and practical procedure to access carbinol derivatives.

## 1. Introduction

Diarylmethanols [1,2] are not only versatile building blocks for organic synthesis, but are also generally used as important precursors for the synthesis of many pharmaceutically active compounds. For example, the 1,1-diarylalkyl moiety is found in compounds with reported activity as antimuscarinics [3], antidepressants [4], and endothelin antagonists [5]. One of the most common methods for the synthesis of these compounds involves the addition reaction of aldehydes with organometallic reagents [6], such as organolithium [7], organomagnesium [8], organotin [9], organoaluminum [10], and organozinc compounds [11,12,13]. Compared to these abovementioned organometallic reagents, organoboron reagents [14,15,16] are highly regarded due to their advantages of stability to air and moisture as well as good functional group tolerance. As a consequence, considerable efforts have been made to develop efficient strategies for the construction of diarylmethanols by the use of organoboron reagents as coupling partners. In 1998, the pioneering work of Miyaura and co-workers [17] on the rhodium-catalyzed addition reaction of arylboronic acids to aldehydes provided attractive and valuable routes for the synthesis of diarylmethanols. Since then, many examples have been reported by other research groups [18,19,20,21,22,23,24,25,26,27,28,29,30,31,32]. Our group also developed a transition-metal-catalyzed reaction of arylboronic acids with aldehydes under different reaction conditions, leading to the selective synthesis of diaryl ketones [33,34], diarylmethanols [35,36] and aryl benzoate derivatives [37]. However, as for most transition-metal-catalyzed reactions using organoboron reagents as coupling partners, the presence of at least stoichiometric amounts of a base was required to accelerate the transmetalation step in the catalytic cycle of the transformation. It was previously reported that the addition of organoboron reagents to aldehydes could proceed readily in the presence of various bases, such as *^t^*BuOK [18,19,20], NaOMe [21], NaOH [22,23], etc. As a result, base-sensitive substrates may be incompatible due to the use of large amounts of a base, especially a strong base [18,19,20,21] which may be a major limitation for these applications. Therefore, the development of an efficient and operationally simple catalyst system avoiding the use of large amounts of a base remains a challenge and has becomes an urgent issue.

**Scheme 1:**
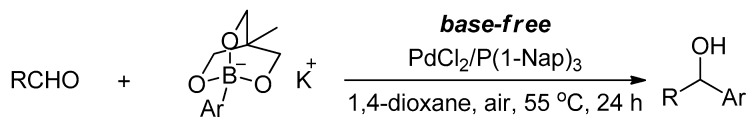
Palladium-catalyzed addition of aryltriolborates to aldehydes affording carbinol derivatives.

To the best of our knowledge, the synthesis of diarylmethanols using aryltriolborates as coupling partners has very few reports to date [32], even though aryltriolborates have exceptionally high levels of stability in air/water, reasonable solubility in organic solvents, and are generally used as the aryl source for C-C and C-N bond-forming reactions [38,39,40,41,42,43]. As part of the continuing efforts in our laboratory toward the development of palladium-catalyzed addition reactions [33,34,35,36,37,44,45,46,47,48,49,50,51,52], we herein report a simple and efficient protocol for the synthesis of diarylmethanols by the Pd-catalyzed base-free addition of aryltriolborates to aldehydes (Scheme 1).

## 2. Results and Discussion

We began our investigation by examining the reaction between *p*-methoxybenzaldehyde (**1b**) and phenyltriolborate (**2a**) to obtain the optimal reaction conditions (Table 1).

Through a screening process, no target product was detected using the common PPh_3_ as a ligand with a variety of parameters including palladium sources, solvents, and temperature. To our delight, a trace amount of the desired product (4-methoxyphenyl)(phenyl)methanol (**3b**) was observed by GC/MS (EI) analysis using tris(2,6-dimethylphenyl)phosphine as the ligand in the presence of PdCl_2_ (Table 1, entry 1). Moreover, we were pleased to find that the yield of the desired product **3b** could be improved to 18% in THF under an air atmosphere after the ligand was changed to the bulky, electron-rich tri(naphthalen-1-yl)phosphine [P(1-Nap)_3_] (Table 1, entry 2). Encouraged by this promising result, a series of trial experiments was performed in the presence of palladium catalysts and with adjustments to the reaction parameters in order to obtain more satisfactory results. First, we investigated the solvent effect and found that 1,4-dioxane was superior to other solvents such as THF, toluene, xylene, *n*-hexane, ethyl acetate, isopropanol, and DMF (Table 1, entries 2–9). Among the palladium sources used, PdCl_2_ exhibited the highest catalytic reactivity in 87% yield (Table 1, entries 8 and 10–19). Increasing or decreasing the amount of P(1-Nap)_3_ in the system affected the yield of the reaction to some extent (Table 1, entries 20–22). In addition, the desired product **3b** was isolated in 85% yield when the procedure was carried out under a N_2_ atmosphere (Table 1, entry 23).

With the optimized reaction conditions in hand, we next examined the scope and generality of the addition reaction of various aldehydes with aryltriolborates.

First, the addition reaction between various aldehydes (**1a**–**1o**) with phenyltriolborate (**2a**) was investigated under standard conditions, and the results are summarized in Table 2. The mono-substituent positions at the phenyl moiety of aromatic aldehydes were evaluated, and the results demonstrated that steric effects of substituents had little impact on the yield of the reaction. For example, the addition reaction of **2a** with *para*-, and *ortho*-methoxybenzaldehyde was examined, and 87% of **3b** and 84% of **3c** were isolated (Table 2, entries 2 and 3). The same phenomenon was observed in the addition reaction of **2a** with *para*-, *meta*- and *ortho*-nitrobenzaldehyde (**1d**–**1f**) to afford **3d**, **3e,** and **3f** in 96%, 99%, and 94% isolated yield, respectively (Table 2, entries 4–6). The electronic properties of the substituents on the phenyl ring of the aromatic aldehydes affected the yields of the reaction to some extent. In general, the aromatic aldehydes bearing an electron-withdrawing substituent (e.g., –F, –Cl, –NO_2_ and –CN) produced a slightly higher yield of products than those analogues bearing an electron-donating substituent (e.g., –OMe) (Table 2, entries 2, 4, 7, 9, and 10). It is noteworthy that substrate **1h**, bearing two functionalized formyl groups, was treated with **2a** to afford the corresponding product **3h,** leaving a formyl group on the 4-position untouched (Table 2, entry 8) [30,35], which may be due to the electronic nature playing important roles, and the activity of carbonyl was decreased in the product **3h**, and hampered the further addition of **2a** to the formyl group of **3h**. Substrate **1k**, bearing a naphthyl group, was treated with **2a** to deliver the desired product **3k** in 83% yield (Table 2, entry 11). Gratifyingly, substrates **1l**–**1m**, bearing a heteroaryl group, underwent the reaction smoothly to afford the target products **3l** and **3m** in 83% and 86% yields, respectively (Table 2, entries 12 and 13). On the other hand, the transformation of aliphatic aldehydes, such as 3-phenylpropanal (**1n**) and butyraldehyde (**1o**), with **2a** also proceeded successfully to provide the corresponding products **3n** and **3o** in 87% and 84% yields, respectively (Table 2, entries 14 and 15). Unfortunately, aromatic ketones and α,β-unsaturated aldehydes cannot proceed smoothly under present condition (Table 2, entries 16 and 17).

Next, we turned our attention to the effect of the reaction of various aryltriolborates (**2a**–**2k**) with benzaldehyde (**1a**) or *p*-nitrobenzaldehyde (**1d**) under standard conditions (Table 3). As expected, the groups on the phenyl ring of aryltriolborates, such as methyl, methoxy, fluoro, chloro, naphthyl, and thienyl, were quite compatible under the optimized reaction conditions. The electronic properties of the groups on the phenyl moiety of aryltriolborates had little effect on the reaction. For example, substrate **2f** bearing an electron-donating substituent (e.g., –OMe), or substrate **2g** bearing an electron-withdrawing substituent (e.g., –F), reacted with **1d** smoothly and afforded the corresponding products **3t** and **3u** in 99% and 96% yields, respectively (Table 3, entries 7 and 8). It is noteworthy that the chloro (commonly used for cross-coupling reaction), and fluoro moieties in aromatic aldehydes were all tolerated and afforded several halogen-containing products **3u**–**3v** in excellent yields (Table 3, entries 8 and 9). Moreover, bicycloltriolborates such as biphenyl-4-yltriolborate (**2i**) and naphthalen-1-yltriolborate (**2j**) were also good partners and coupled with **1d** efficiently, affording the corresponding products **3w**–**3x** in good yields (Table 3, entries 10 and 11). Heterocyclic triolborates, and in particular 3-thienyltriolborate (**2k**) is a good partner for this transformation, and the desired product **3y** was isolated in 85% yield (Table 3, entry 12). However, the treatment of an alkyltriolborate such as methyltriolborate or cyclopropyltriolborate with **1d** under the optimized conditions afforded only a trace amount of the desired products.

**Scheme 2:**
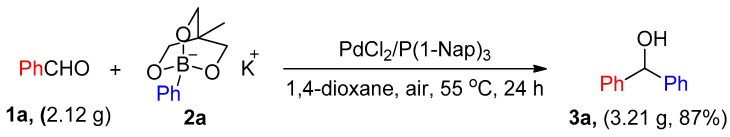
Gram-scale synthesis of diphenylmethanol (**3a**).

Finally, the present synthetic route to diarylmethanols could be readily scaled up to gram quantity without difficulty. For instance, the reaction at the 20 mmol scale afforded the corresponding product diphenylmethanol (**3a**) in 87% yield (Scheme 2).

**Scheme 3:**
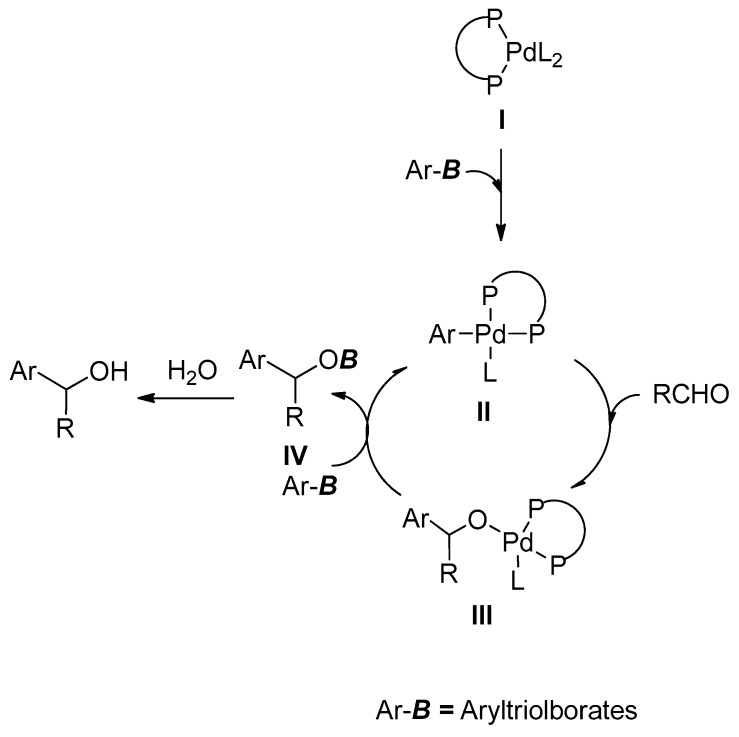
Tentative mechanism for the formation of carbinol.

A tentative reaction mechanism is illustrated in Scheme 3. It has been suggested that the transmetalation of an electrophilic Pd(II) intermediate such as **I** can lead to intermediate **II**. Coordination of the aldehyde to the electrophilic metal center followed by the migration of the aryl group to the activated aldehyde then leads to intermediate **III,** from which the carbinol is released and the catalyst is regenerated.

## 3. Materials and Methods

### 3.1. General Information

Chemicals were received from commercial sources without further purification, or prepared by methods from the literature. ^1^H-NMR and ^13^C-NMR spectra were measured on a 300 or 500 MHz Bruker (Billerica, MA, USA) spectrometer, using CDCl_3_ as the solvent with tetramethylsilane (TMS) as the internal standard at room temperature. Chemical shifts are given in *δ* relative to TMS; the coupling constants *J* are given in Hz. All reactions were conducted under air atmosphere. Column chromatography was performed using EM Silica gel 60 (300–400 mesh). All products are known compounds and identified by comparison with authentic samples. The structures of all the title compounds **3a**–**3y** were characterized by ^1^H-NMR and ^13^C-NMR spectra (Supplementary Materials).

### 3.2. General Procedure for the Synthesis of Carbinol Derivatives through the Palladium-Catalyzed Addition of Aryltriolborates to Aldehydes

Under air atmosphere, a Teflon-valve-sealed Schlenk tube was charged with aldehydes (0.3 mmol), aryltriolborates (0.6 mmol), PdCl_2_ (5 mol %) and P(1-Nap)_3_ (5 mol %) in 1,4-dioxane (3 mL) at room temperature. The reaction mixture was stirred vigorously at 55 °C for 24 h. After the completion of the reaction, as monitored by TLC and GC-MS analysis, the reaction mixture was cooled to room temperature. The mixture was extracted with diethyl ether (3 × 10 mL). The combined ether extracts were concentrated in vacuo and the residue was purified by flash column chromatography on silica gel to give the desired product carbinol derivatives.

*Diphenylmethanol* (**3a**): While solid; m.p. 67–68 °C (Lit. not reported); ^1^H-NMR (CDCl_3_, 300 MHz): δ 2.28 (s, 1H), 5.85 (s, 1H), 7.25–7.30 (m, 2H), 7.32–7.41 (m, 8H); ^13^C-NMR (CDCl_3_, 125 MHz): δ 76.2, 126.5, 127.5, 128.5, 143.8.

*(4-Methoxyphenyl)(phenyl)methanol* (**3b**): Oil; ^1^H-NMR (CDCl_3_, 500 MHz): δ 2.42 (s, 1H), 3.76 (s, 3H), 5.75 (s, 1H), 6.83–6.85 (m, 2H), 7.23–7.26 (m, 3H), 7.31–7.36 (m, 4H); ^13^C-NMR (CDCl_3_, 125 MHz): δ 55.2, 75.7, 113.8, 126.3, 127.3, 127.9, 128.4, 136.1, 144.0, 158.9.

*(2-Methoxyphenyl)(phenyl)methanol* (**3c**): Oil; ^1^H-NMR (CDCl_3_, 500 MHz): δ 3.09 (s, 1H), 3.80 (s, 3H) 6.05–6.06 (m, 1H), 6.88–6.96 (m, 2H), 7.22–7.39 (m, 7H); ^13^C-NMR (CDCl_3_, 125 MHz): δ 55.4, 72.2, 110.7, 120.8, 126.5, 127.1, 127.8, 128.1, 128.6, 129.5, 143.2, 156.7.

*(4-Nitrophenyl)(phenyl)methanol* (**3d**): Pale yellow solid; m.p. 70.2–71.3 °C (Lit. 72 °C); ^1^H-NMR (CDCl_3_, 500 MHz): δ 2.80 (s, 1H), 5.88 (s, 1H), 7.29–7.37 (m, 5H), 7.55 (d, *J* = 8.5 Hz, 2H), 8.15 (d, *J* = 9.0 Hz, 2H); ^13^C-NMR (CDCl_3_, 125 MHz): δ 75.5, 123.7, 126.7, 127.1, 128.4, 129.0, 142.7, 147.1, 150.9.

*(3-Nitrophenyl)(phenyl)methanol* (**3e**): Oil; ^1^H-NMR (CDCl_3_, 500 MHz): δ 2.75 (s, 1H), 5.89 (s, 1H), 7.29–7.38 (m, 5H), 7.48 (t, *J* = 8.0 Hz, 1H), 7.69 (d, *J* = 8.0 Hz, 1H), 8.09 (d, *J* = 8.0 Hz, 1H), 8.27 (s, 1H); ^13^C-NMR (CDCl_3_, 125 MHz): δ 75.4, 121.3, 122.4, 126.67, 128.4, 128.9, 129.4, 132.5, 142.8, 145.8, 148.3.

*(2-Nitrophenyl)(phenyl)methanol* (**3f**): Oil; ^1^H-NMR (CDCl_3_, 500 MHz): δ 2.96 (s, 1H), 6.42 (s, 1H), 7.28–7.34 (m, 5H), 7.44 (t, *J* = 7.5 Hz, 1H), 7.63 (t, *J* = 7.5 Hz, 1H), 7.74 (d, *J* = 7.5 Hz, 1H), 7.92 (d, *J* = 8.0 Hz, 1H); ^13^C-NMR (CDCl_3_, 125 MHz): δ 71.5, 124.7, 127.0, 128.1, 128.5, 128.6, 129.4, 133.4, 138.5, 141.6, 148.4.

*(4-Cyanophenyl)(phenyl)methanol* (**3g**): Oil; ^1^H-NMR (CDCl_3_, 500 MHz): δ 2.64 (s, 1H), 5.84 (s, 1H), 7.29–7.37 (m, 5H), 7.50 (d, *J* = 8.0 Hz, 2H), 7.60 (d, *J* = 8.5 Hz, 2H); ^13^C-NMR (CDCl_3_, 125 MHz): δ 75.5, 111.1, 118.7, 126.6, 127.0, 128.2, 128.8, 132.2, 142.8, 148.9.

*4-(Hydroxy(phenyl)methyl)benzaldehyde* (**3h**): Oil; ^1^H-NMR (CDCl_3_, 500 MHz): δ 2.54 (s, 1H), 5.89 (s, 1H), 7.29–7.31 (m, 1H), 7.33–7.37 (m, 4H), 7.57 (d, *J* = 8.0 Hz, 2H), 7.84 (d, *J* = 8.0 Hz, 2H), 9.97 (s, 1H); ^13^C-NMR (CDCl_3_, 125 MHz): δ 75.9, 126.7, 126.9, 128.1, 128.8, 129.9, 135.6, 143.1, 150.4, 191.9.

*(4-Fluorophenyl)(phenyl)methanol* (**3i**): Oil; ^1^H-NMR (CDCl_3_, 500 MHz): δ 2.57 (s, 1H), 5.78 (s, 1H), 7.00–7.05 (m, 2H), 7.28–7.38 (m, 7H); ^13^C-NMR (CDCl_3_, 125 MHz): δ 75.5, 115.1, 115.3, 126.4, 127.7, 128.1, 128.2, 128.5, 139.5, 143.6, 161.1, 163.1.

*(4-Chlorophenyl)(phenyl)methanol* (**3j**): Oil; ^1^H-NMR (CDCl_3_, 300 MHz): δ 2.42 (s, 1H), 5.79 (s, 1H), 7.28–7.35 (m, 9H); ^13^C-NMR (CDCl_3_, 125 MHz): δ 75.6, 126.5, 127.8, 127.9, 128.6, 128.6, 133.2, 142.2, 143.4.

*Naphthalen-1-yl(phenyl)methanol* (**3k**): Oil; ^1^H-NMR (CDCl_3_, 500 MHz): δ 2.42 (s, 1H), 6.54 (s, 1H), 7.27–7.35 (m, 3H), 7.41–7.51 (m, 5H), 7.64 (d, *J* = 7.0 Hz, 1H), 7.82–7.89 (m, 2H), 8.05 (d, *J* = 7.5 Hz, 1H); ^13^C-NMR (CDCl_3_, 125 MHz): δ 73.7, 124.0, 124.7, 125.4, 125.6, 126.2, 127.1, 127.7, 128.5, 128.6, 128.8, 130.7, 134.0, 138.8, 143.2.

*Phenyl(thiophen-2-yl)methanol* (**3l**): Oil; ^1^H-NMR (CDCl_3_, 500 MHz): δ 2.51 (s, 1H), 6.06 (s, 1H), 6.89–6.96 (m, 2H), 7.26–7.47 (m, 6H); ^13^C-NMR (CDCl_3_, 125 MHz): δ 72.4, 124.9, 125.4, 126.3, 126.6, 128.0, 128.5, 143.1, 148.1.

*Phenyl(pyridin-4-yl)methanol* (**3m**): While solid; m.p. 123–124 °C (Lit. not reported); ^1^H-NMR (CDCl_3_, 500 MHz): δ 4.87 (s, 1H), 5.74 (s, 1H), 7.27–7.32 (m, 7H), 8.32 (s, 2H); ^13^C-NMR (CDCl_3_, 125 MHz): δ 74.6, 121.4, 126.8, 128.0, 128.7, 143.0, 149.1, 153.4.

*1,3-Diphenylpropan-1-ol* (**3n**): Oil; ^1^H-NMR (CDCl_3_, 500 MHz): δ 2.03–2.17 (m, 3H), 2.67–2.78 (m, 2H), 4.68–4.71 (m, 1H), 7.20–7.23 (m, 3H), 7.29–7.33 (m, 3H), 7.36–7.40 (m, 4H); ^13^C-NMR (CDCl_3_, 125 MHz): δ 32.0, 40.4, 73.8, 125.8, 125.9, 127.6, 128.3, 128.4, 128.5, 141.8, 144.5.

*1-Phenylbutan-1-ol* (**3o**): Oil; ^1^H-NMR (CDCl_3_, 300 MHz): δ 0.90 (t, *J* = 7.5 Hz, 3H), 1.23–1.44 (m, 2H), 1.60–1.80 (m, 2H), 1.97 (s, 1H), 4.63 (d, *J* = 6.0 Hz, 1H), 7.22–7.31 (m, 5H), 7.53 (m, 5H); ^13^C-NMR (CDCl_3_, 125 MHz): δ 13.9, 19.0, 41.2, 74.4, 125.9, 127.4, 128.4, 144.9.

*Phenyl(m-tolyl)methanol* (**3p**): Oil; ^1^H-NMR (CDCl_3_, 500 MHz): δ 2.92 (s, 3H), 2.38 (s, 1H), 5.78 (s, 1H), 7.06 (d, *J* = 5.0 Hz, 1H), 7.13–7.26 (m, 4H), 7.30–7.37 (m, 4H); ^13^C-NMR (CDCl_3_, 125 MHz): δ 21.5, 76.3, 123.7, 126.6, 127.3, 127.5, 128.4, 128.4, 128.5, 138.2, 143.8, 143.9.

*(3-Methoxyphenyl)(phenyl)methanol* (**3q**): Oil; ^1^H-NMR (CDCl_3_, 300 MHz): δ 2.32 (s, 1H), 3.82 (s, 3H), 5.84 (s, 1H), 6.85 (d, *J* = 11 Hz, 1H), 6,99 (d, *J* =11 Hz, 2H), 7.29–7.43 (m, 6H); ^13^C-NMR (CDCl_3_, 125 MHz): δ 55.2, 76.2, 112.1, 113.0, 118.9, 126.5, 127.6, 128.5, 129.5, 143.7, 145.5, 159.8.

*(3-Chlorophenyl)(phenyl)methanol* (**3r**): Oil; ^1^H-NMR (CDCl_3_, 500 MHz): δ 2.46 (s, 1H), 5.78 (s, 1H), 7.23–7.25 (m, 3H), 7.28–7.36 (m, 5H), 7.40 (s, 1H); ^13^C-NMR (CDCl_3_, 125 MHz): δ 75.7, 124.7, 126.6, 126.6, 126.7, 128.0, 128.7, 129.8, 134.4, 143.2, 145.8.

*(4-Nitrophenyl)(p-tolyl)methanol* (**3s**): Pale yellow solid; m.p. 99–100 °C (Lit. 97 °C); ^1^H-NMR (CDCl_3_, 500 MHz): δ 2.34 (s, 3H), 2.51 (s, 1H), 5.87 (s, 1H), 7.16 (d, *J* = 8.0 Hz, 2H), 7.22 (d, *J* = 8.0 Hz, 2H), 7.56 (d, *J* = 8.5 Hz, 2H), 8.17 (d, *J* = 8.5 Hz, 2H); ^13^C-NMR (CDCl_3_, 125 MHz): δ 21.1, 75.3, 123.6, 126.7, 127.0, 129.6, 138.3, 139.8, 147.1, 151.0.

*(4-Methoxyphenyl)(4-nitrophenyl)methanol* (**3t**): Pale yellow solid; m.p. 57–58 °C (Lit. 55.4–56.8 °C); ^1^H-NMR (CDCl_3_, 500 MHz): δ 2.55 (s, 1H), 3.70 (s, 3H), 5.86 (s, 1H), 6.87 (d, *J* = 9.0 Hz, 2H), 7.23 (d, *J* = 9.0 Hz, 2H), 7.55 (d, *J* = 9.0 Hz, 2H), 8.16 (d, *J* = 9.0 Hz, 2H); ^13^C-NMR (CDCl_3_, 125 MHz): δ 55.3, 75.2, 114.2, 123.6, 126.9, 128.1, 135.0, 147.0, 151.1, 159.6.

*(4-Fluorophenyl)(4-nitrophenyl)methanol* (**3u**): Pale yellow solid; m.p. 75–76 °C (Lit. 77 °C); ^1^H-NMR (CDCl_3_, 400 MHz): δ 2.17 (s, 1H), 5.92 (s, 1H), 7.05 (t, *J* = 8.8 Hz, 2H), 7.34–7.31 (m, 2H), 7.56 (d, *J* = 8.8 Hz, 2H), 8.19 (d, *J* = 8.8 Hz, 2H); ^13^C-NMR (CDCl_3_, 125 MHz): δ 74.7, 115.7, 115.8, 123.7, 127.0, 128.4, 128.5, 138.5, 138.5, 147.2, 150.6, 161.5, 163.5.

*(4-Chlorophenyl)(4-nitrophenyl)methanol* (**3v**): Pale yellow solid; m.p. 133–134 °C (Lit. 132–133 °C); ^1^H-NMR (CDCl_3_, 500 MHz): δ 2.68 (s, 1H), 5.88 (s, 1H), 7.27 (d, *J* = 8.5 Hz, 2H), 7.32 (d, *J* = 8.5 Hz, 2H), 7.53 (d, *J* = 9.0 Hz, 2H), 8.17 (d, *J* = 8.5 Hz, 2H); ^13^C-NMR (CDCl_3_, 125 MHz): δ 74.8, 123.7, 127.0, 128.0, 129.0, 134.2, 141.1, 147.3, 150.3

*Biphenyl-4-yl(4-nitrophenyl)methanol* (**3w**): While solid; m.p. 138.1–139 °C (Lit. 138.7–140.1 °C); ^1^H-NMR (CDCl_3_, 300 MHz): δ 2.55 (s, 1H), 5.96 (s, 1H), 7.34–7.47 (m, 5H), 7.55–7.63 (m, 6H), 8.20 (d, *J* = 8.4 Hz, 2H); ^13^C-NMR (CDCl_3_, 125 MHz): δ 75.3, 123.7, 127.0, 127.1, 127.1, 127.5, 127.6, 128.8, 140.3, 141.3, 141.6, 147.2, 150.7.

*Naphthalen-1-yl(4-nitrophenyl)methanol* (**3x**): Oil; ^1^H-NMR (CDCl_3_, 500 MHz): δ 2.92 (s, 1H), 6.49 (s, 1H), 7.44–7.50 (m, 4H), 7.53 (d, *J* = 8.5 Hz, 2H), 7.82–7.88 (m, 2H), 7.99 (d, *J* = 8.0 Hz, 1H), 8.11 (d, *J* = 9.0 Hz, 2H); ^13^C-NMR (CDCl_3_, 125 MHz): δ 73.0, 123.4, 123.5, 125.1, 125.4, 125.8, 126.4, 127.2, 128.8, 129.1, 130.3, 134.0, 137.6, 147.0, 150.2.

*(4-Nitrophenyl)(thiophen-3-yl)methanol* (**3y**): Pale yellow solid; m.p. 99–100 °C; ^1^H-NMR (CDCl_3_, 500 MHz): δ 2.56 (s, 1H), 5.99 (s, 1H), 6.97 (d, *J* = 5.0 Hz, 1H), 7.22–7.32 (m, 2H), 7.58 (d, *J* = 8.5 Hz, 2H), 8.19 (d, *J* = 8.5 Hz, 2H); ^13^C-NMR (CDCl_3_, 125 MHz): δ 71.6, 122.5, 123.7, 125.9, 127.0, 129.1, 144.0, 147.3, 150.3.

## 4. Conclusions

In summary, we have developed a base-free protocol for the synthesis of a wide range of diarylmethanols in good to excellent yields via the palladium-catalyzed addition of aryltriolborates to aldehydes. The base-free conditions tolerate a broad range of substrates and functional groups. Further efforts to explore other palladium-catalyzed base-free reactions using aryltriolborates as coupling partners and to extend the applications of the transformation are currently underway in our laboratories.

## Figures and Tables

**Table 1:**
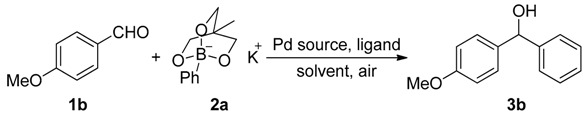
Optimization of the reaction conditions ^a^.

Entry	Pd Source	Pd/L Ratio (x)	Solvent	Yield ^b^ (%)
1 ^c^	PdCl_2_	1	THF	trace
2	PdCl_2_	1	THF	18
3	PdCl_2_	1	Toluene	43
4	PdCl_2_	1	xylene	42
5	PdCl_2_	1	*n*-hexane	71
6	PdCl_2_	1	ethyl acetate	38
7	PdCl_2_	1	isopropanol	trace
8	PdCl_2_	1	1,4-dioxane	87(30) ^e^ (57) ^f^
9	PdCl_2_	1	DMF	trace
10	Pd(OAc)_2_	1	1,4-dioxane	49
11	Pd(TFA)_2_	1	1,4-dioxane	48
12	PdCl_2_(PPh_3_)_2_	1	1,4-dioxane	23
13	PdCl_2_(dppf)	1	1,4-dioxane	61
14	PdCl_2_(dppe)	1	1,4-dioxane	41
15	PdCl_2_(cod)	1	1,4-dioxane	43
16	PdCl_2_(Py)_2_	1	1,4-dioxane	10
17	PdCl_2_(MeCN)_2_	1	1,4-dioxane	29
18	Pd(PPh_3_)_4_	1	1,4-dioxane	38
19	Pd_2_(dba)_3_	1	1,4-dioxane	53
20	PdCl_2_	0.5	1,4-dioxane	68
21	PdCl_2_	1.5	1,4-dioxane	75
22	PdCl_2_	2	1,4-dioxane	79
23	PdCl_2_	1	1,4-dioxane	85 ^d^

^a^ Reaction conditions: **1b** (0.3 mmol), **2a** (0.6 mmol), Pd source (5 mol %), Pd/P(1-Nap)_3_ ratio (x), solvent (3 mL), air, 55 °C, 24 h; ^b^ Isolated yield; ^c^ Using tris(2,6-dimethylphenyl)phosphine as the ligand; ^d^ Under a N_2_ atmosphere; ^e^ 25 °C; ^f^ 12 h at 55 °C.

**Table 2:**
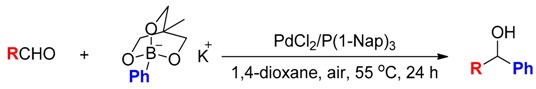
Addition of various aldehydes with phenyltriolborate ^a^.

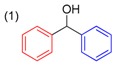	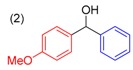	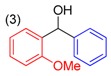	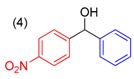	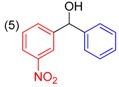
**3a** (94%)	**3b** (87%)	**3c** (84%)	**3d** (96%)	**3e** (99%)
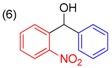	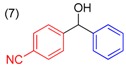	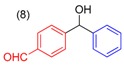	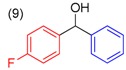	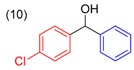
**3f** (94%)	**3g** (95%)	**3h** (72%)	**3i** (95%)	**3j** (91%)
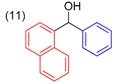	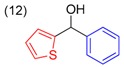	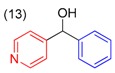	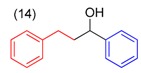	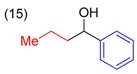
**3k** (83%)	**3l** (83%)	**3m** (86%)	**3n** (87%)	**3o** (84%)
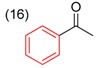	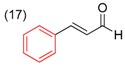			
**1p** (0%)	**1q** (0%)			

^a^ Reaction conditions: **1** (0.3 mmol), **2a** (0.6 mmol), PdCl_2_ (5 mol %), P(1-Nap)_3_ (5 mol %), 1,4-dioxane (3 mL), air, 55 °C, 24 h.

**Table 3:**
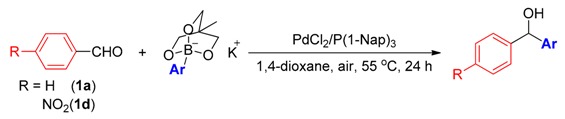
Addition of **1a**/**1d** with various aryltriolborates ^a^.

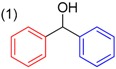	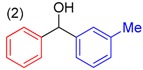	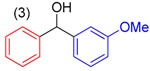	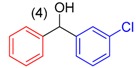
**3a** (94%)	**3p** (90%)	**3q** (95%)	**3r** (91%)
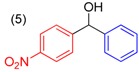	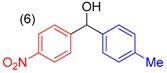	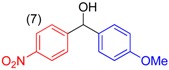	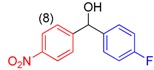
**3d** (96%)	**3s** (91%)	**3t** (99%)	**3u** (96%)
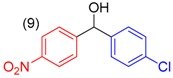	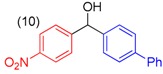	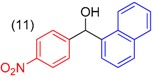	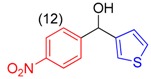
**3v** (97%)	**3w** (93%)	**3x** (89%)	**3y** (85%)

^a^ Reaction conditions: **1** (0.3 mmol), **2a** (0.6 mmol), PdCl_2_ (5 mol %), P(1-Nap)_3_ (5 mol %), 1,4-dioxane (3 mL), air, 55 °C, 24 h.

## Supplementary Materials

Supplementary materials are available online.
