# Supplementary material for: Efficient Approach to Carbinol Derivatives through Palladium-Catalyzed Base-Free Addition of Aryltriolborates to Aldehydes
Source: Molecules. 2017 Sep 20;22(9):1580. doi: 10.3390/molecules22091580 (PMC6151780; doi:10.3390/molecules22091580)
Supplement: Supplementary file 1 [file molecules-22-01580-s001.pdf]

Supporting Information

Base-free efficient approach to carbinol derivatives through palladium-catalyzedaddition of aryltriolborates to aldehydes

Kun Hu, Pengqing Ye, Qianqian Zhen, Xinrong Yao, Tong Xu, Yinlin Shao*

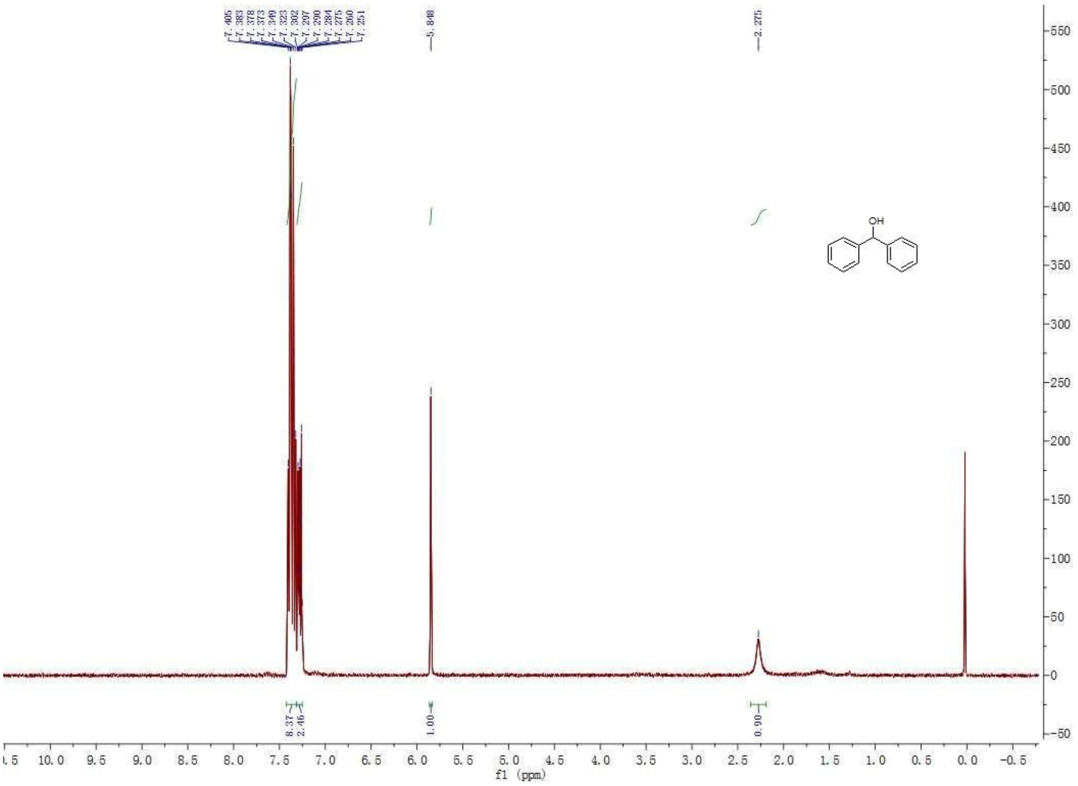

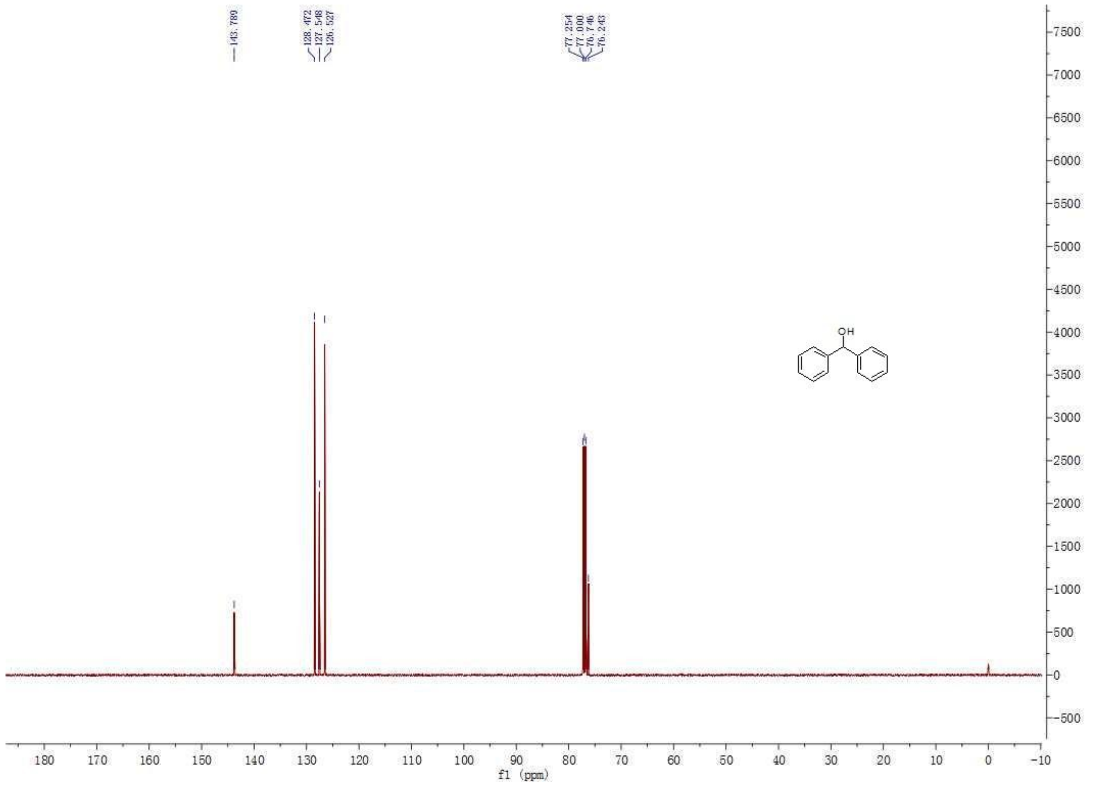

Figure S1. ^{1}H NMR of **3a** (300 MHz, $CDCl_3$) and ^{13}C NMR of **3a** (125 MHz, $CDCl_3$).

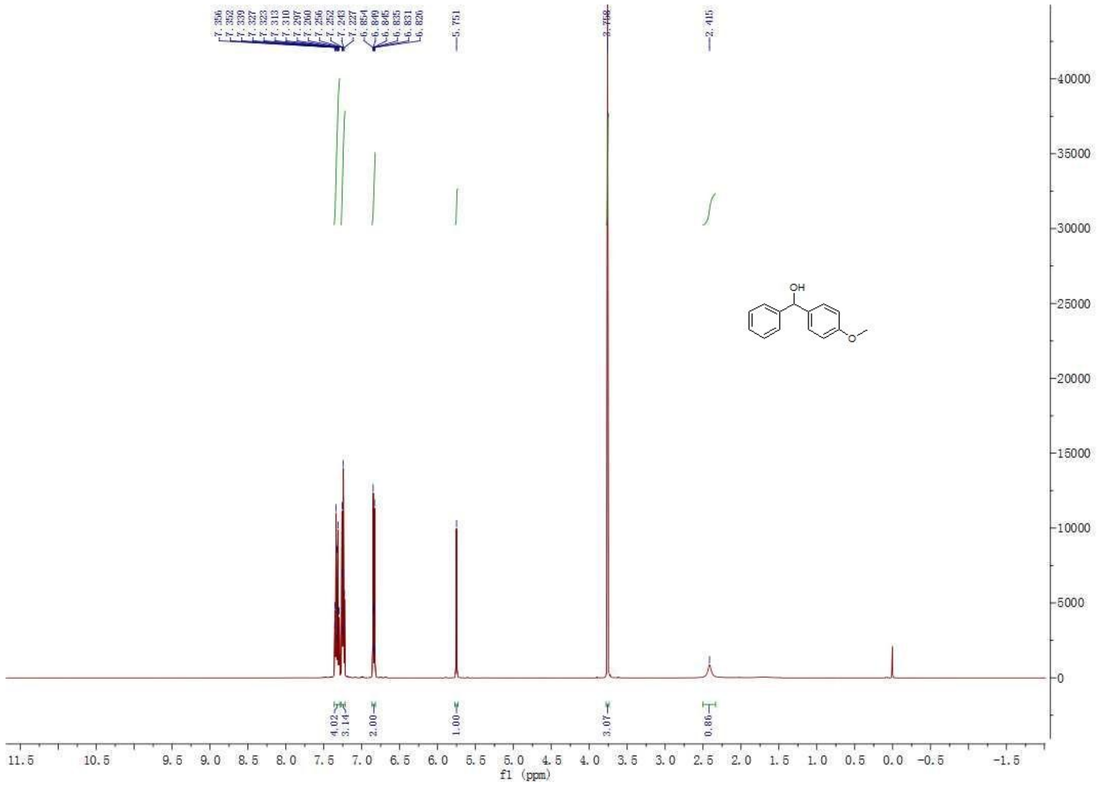

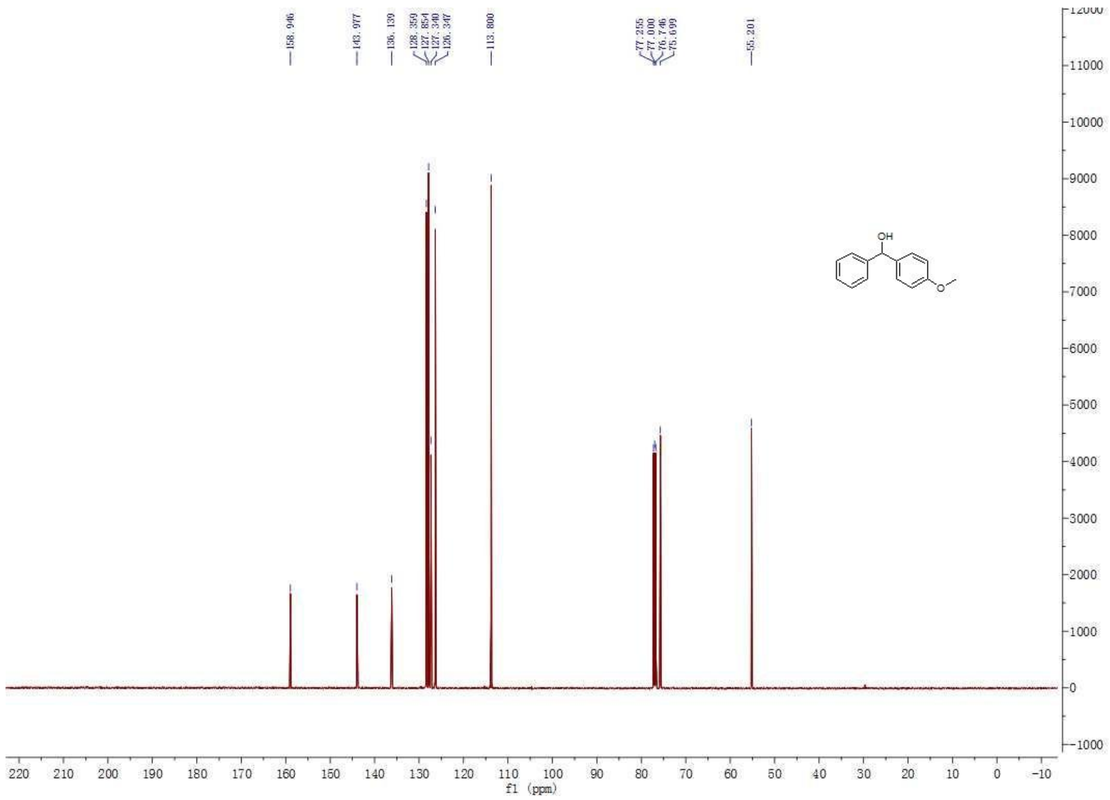

Figure S2. ^{1}H NMR of **3b** (500 MHz, $CDCl_3$) and ^{13}C NMR of **3b** (125 MHz, $CDCl_3$).

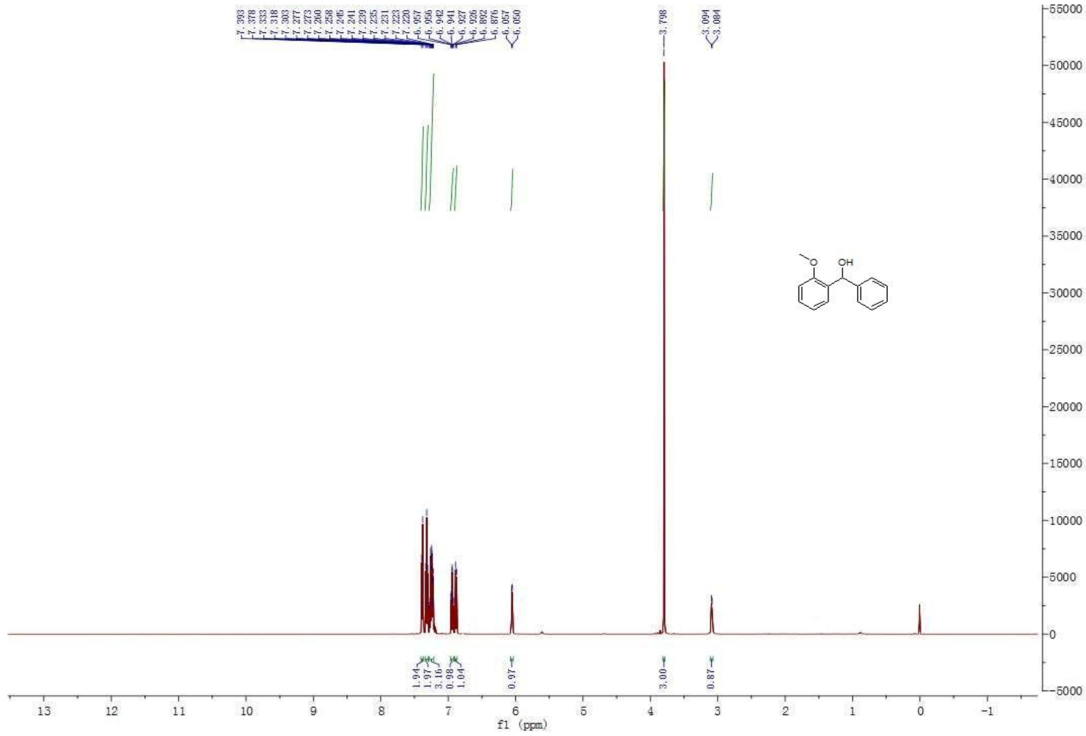

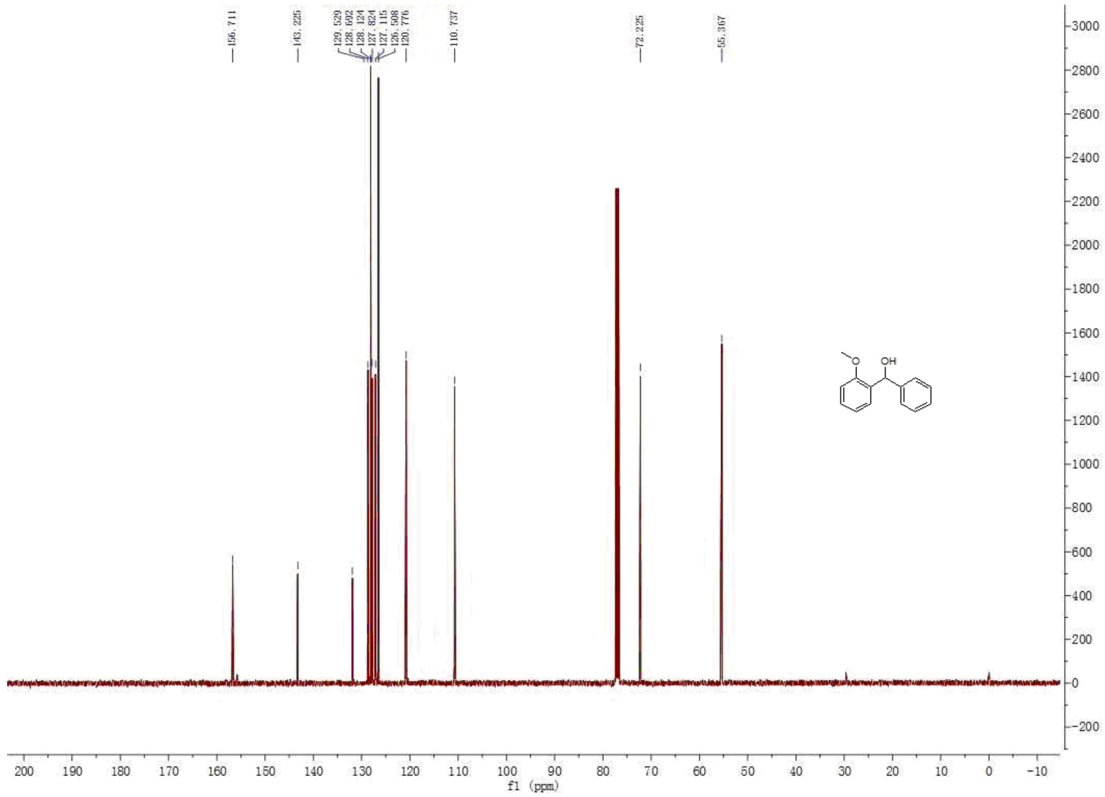

Figure S3. ^{1}H NMR of **3c** (500 MHz, $CDCl_3$) and ^{13}C NMR of **3c** (125 MHz, $CDCl_3$).

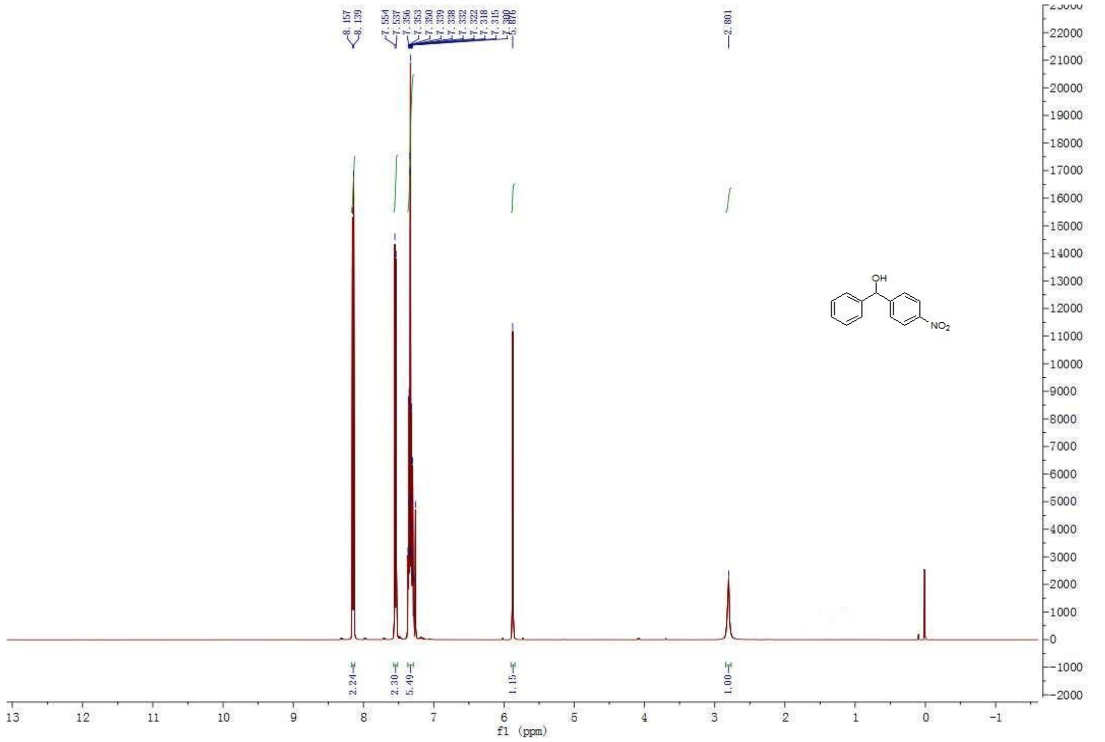

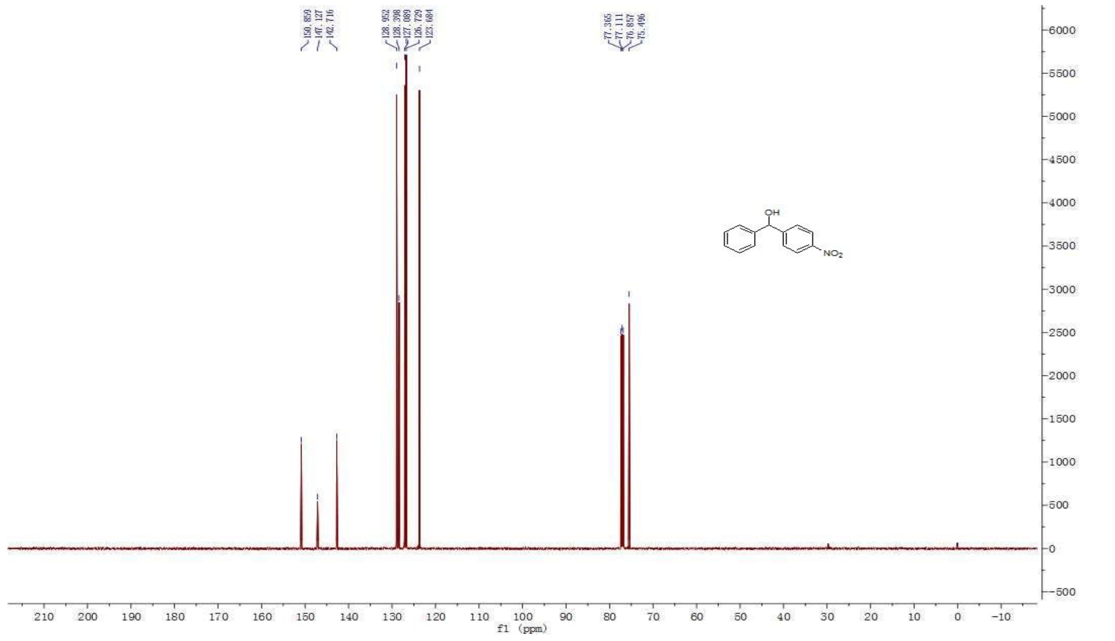

Figure S4. ^{1}H NMR of **3d** (500 MHz, $CDCl_3$) and ^{13}C NMR of **3d** (125 MHz, $CDCl_3$).

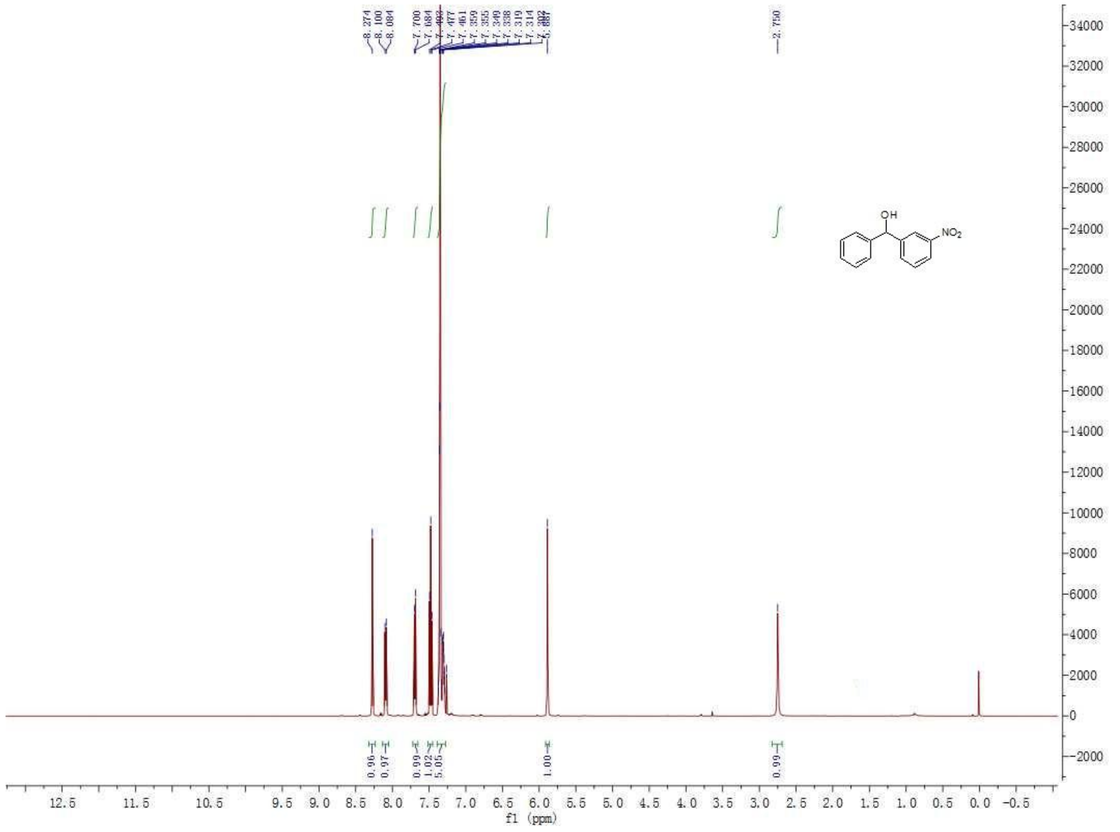

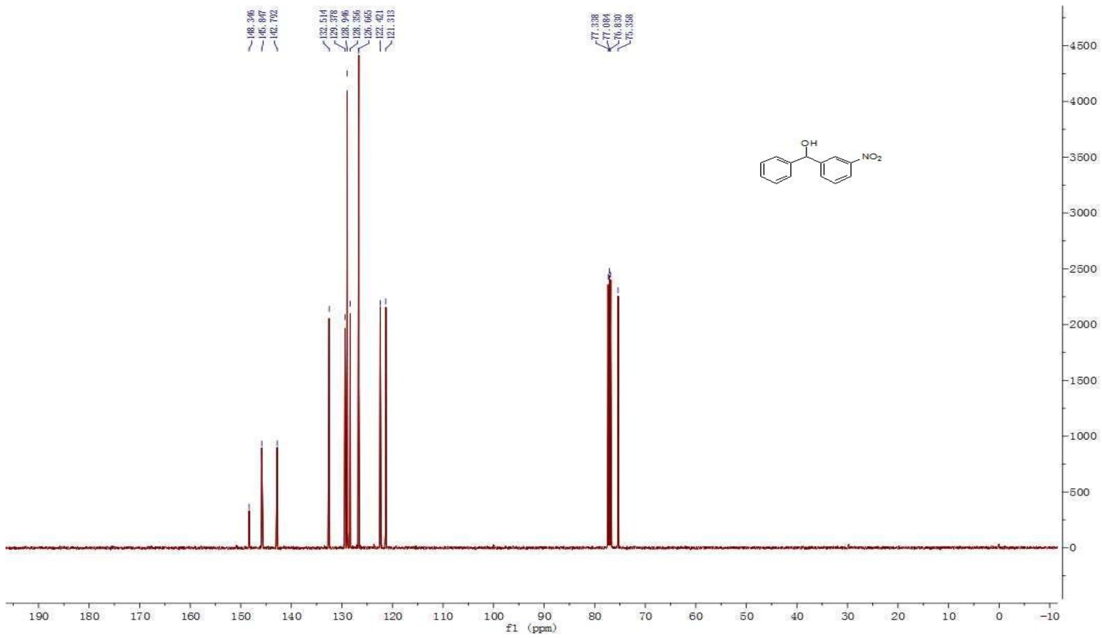

Figure S5. ^{1}H NMR of **3e** (500 MHz, $CDCl_3$) and ^{13}C NMR of **3e** (125 MHz, $CDCl_3$).

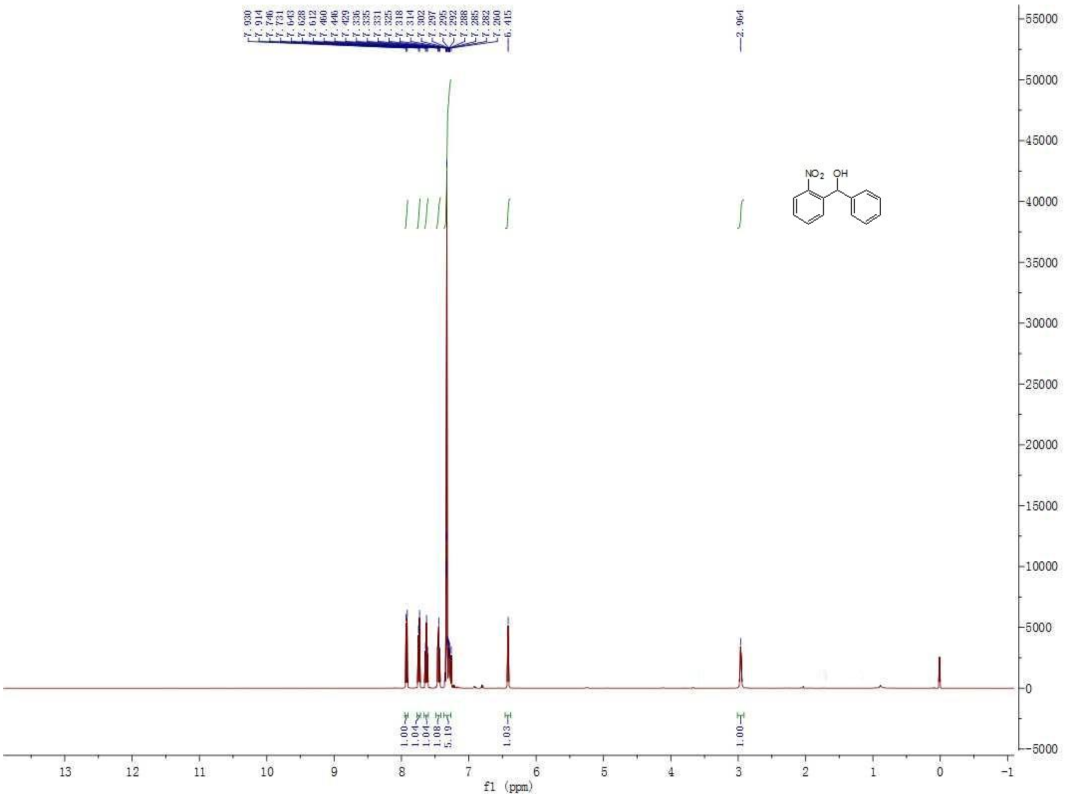

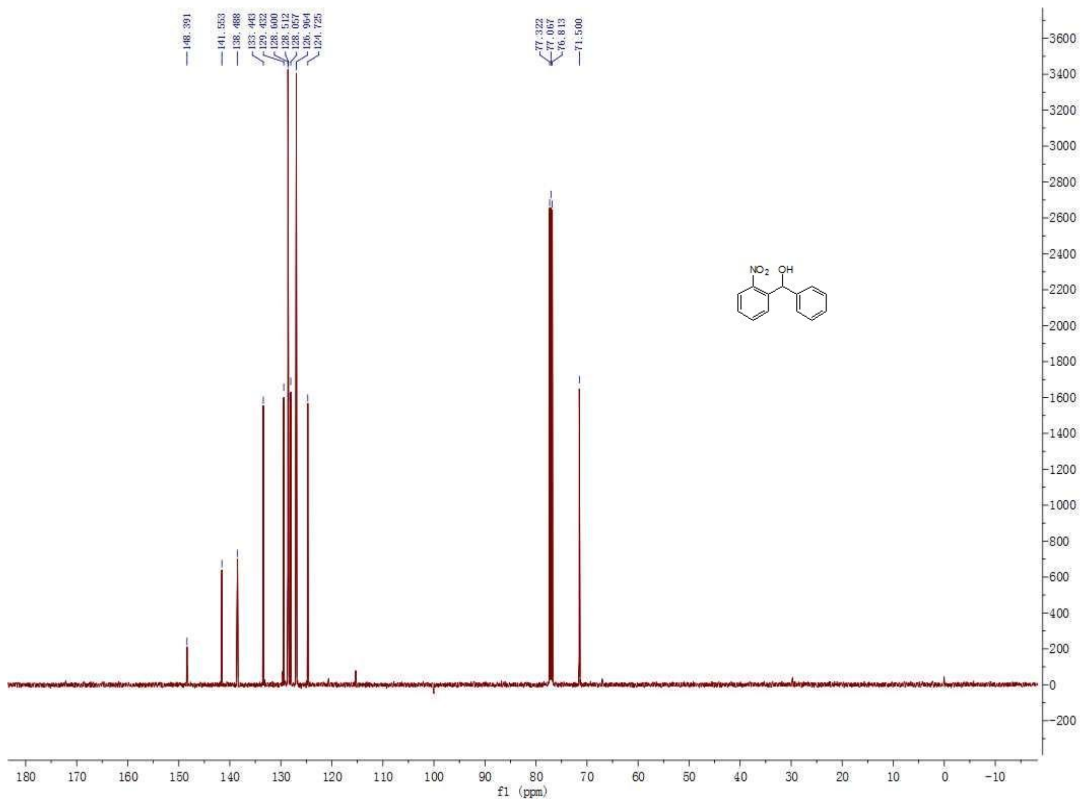

Figure S6. ^{1}H NMR of **3f** (500 MHz, $CDCl_3$) and ^{13}C NMR of **3f** (125 MHz, $CDCl_3$).

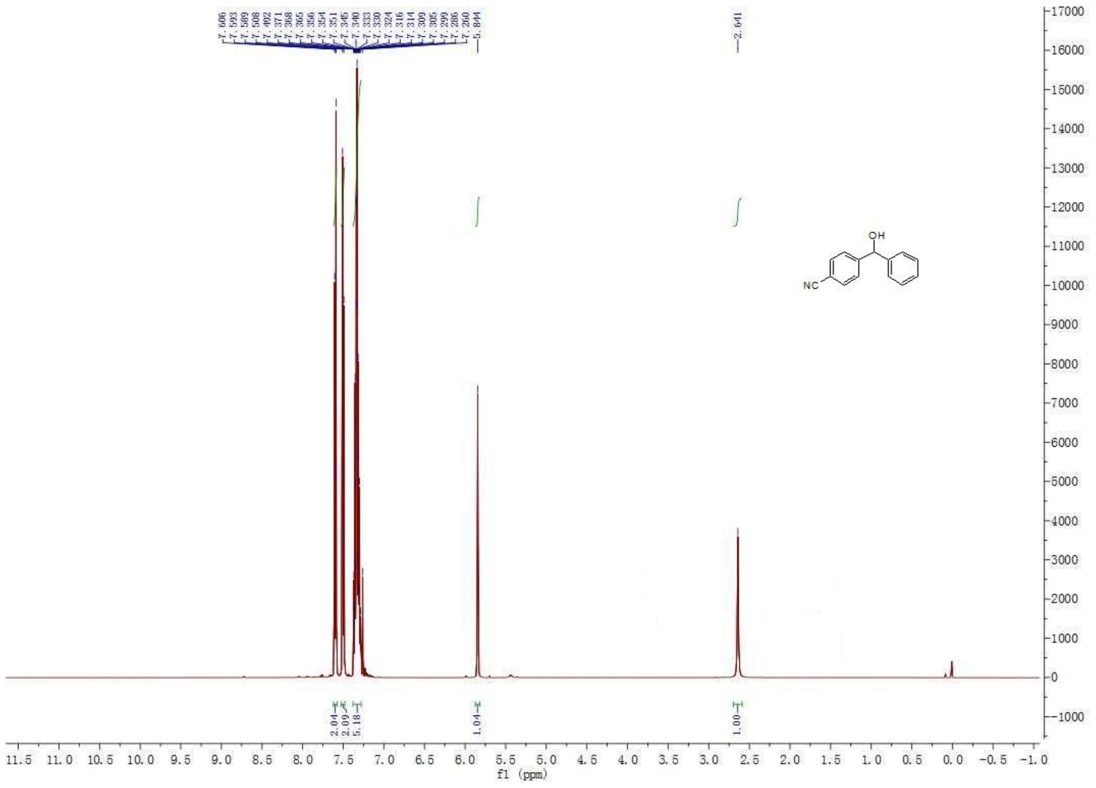

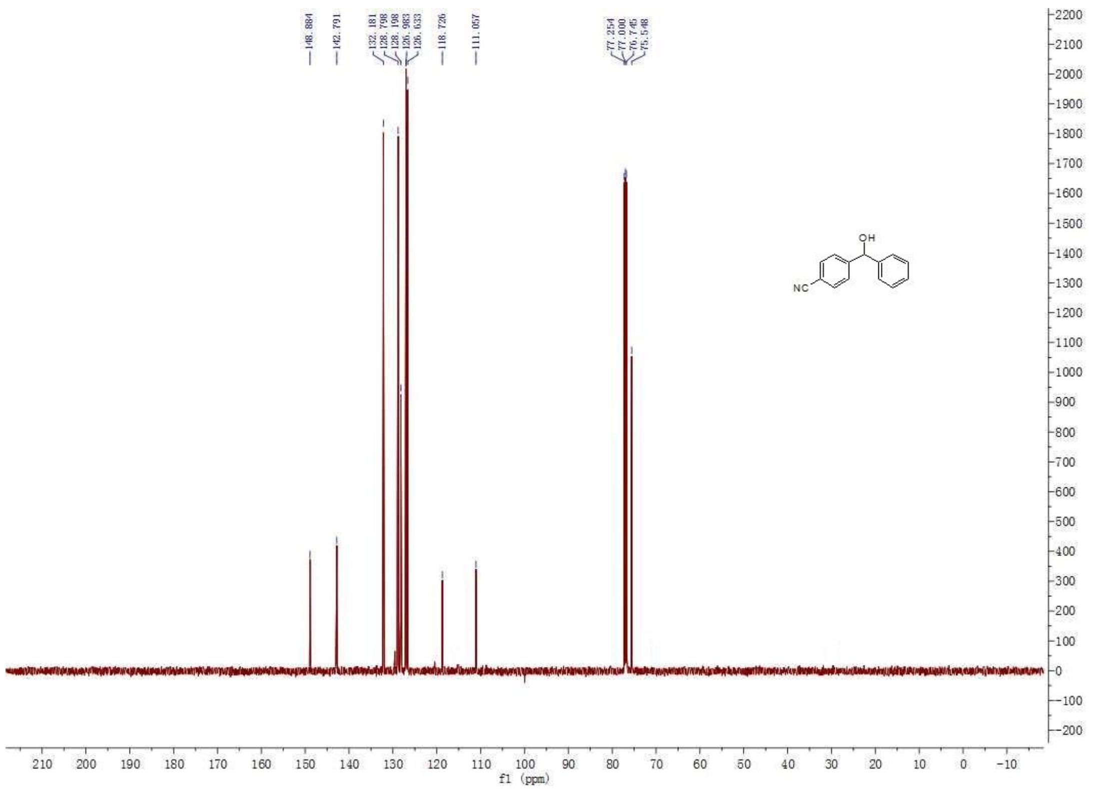

Figure S7. ^{1}H NMR of **3g** (500 MHz, $CDCl_3$) and ^{13}C NMR of **3g** (125 MHz, $CDCl_3$).

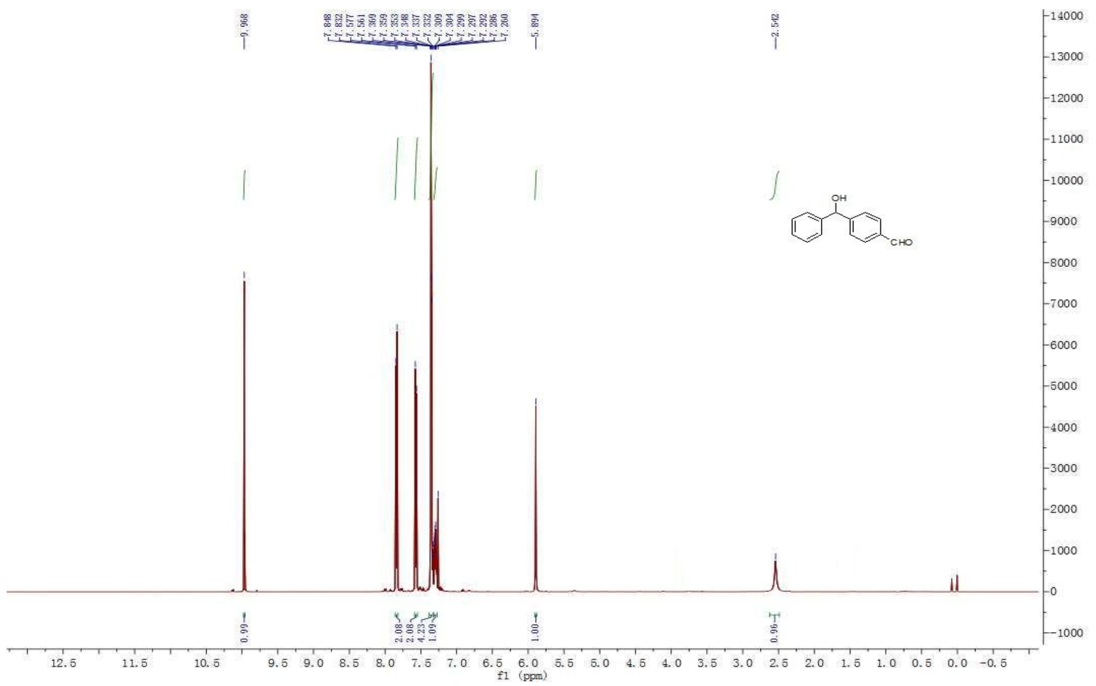

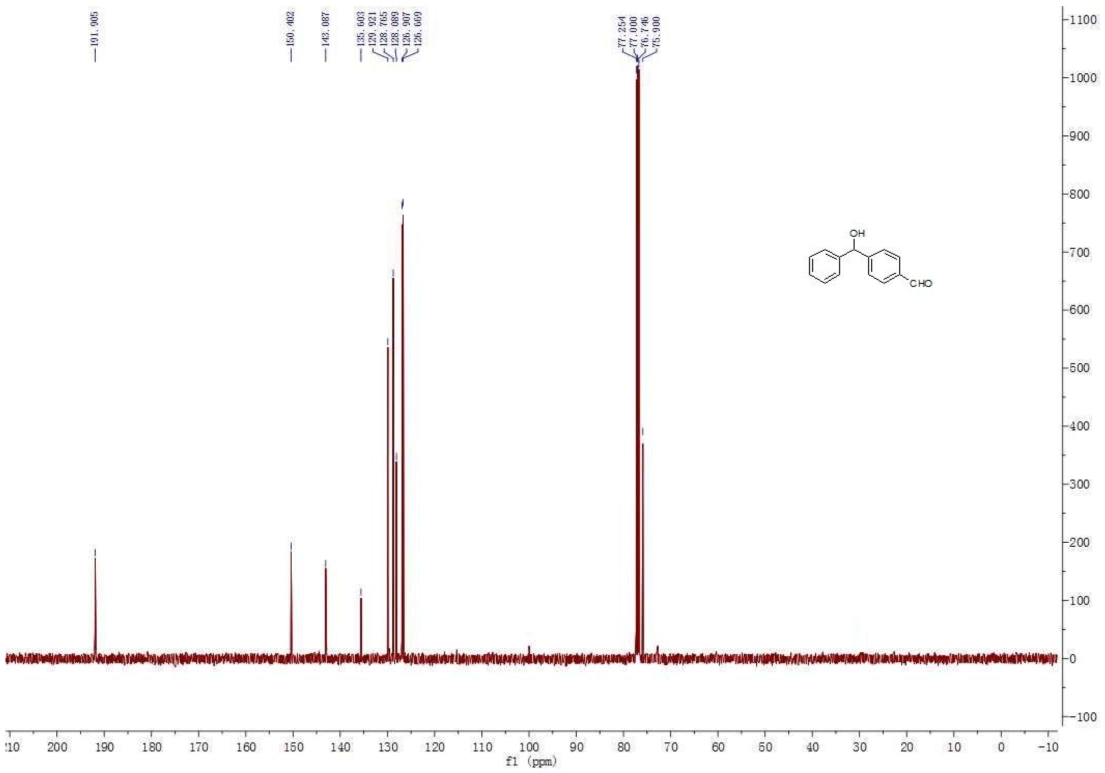

Figure S8. ^{1}H NMR of **3h** (500 MHz, $CDCl_3$) and ^{13}C NMR of **3h** (125 MHz, $CDCl_3$).

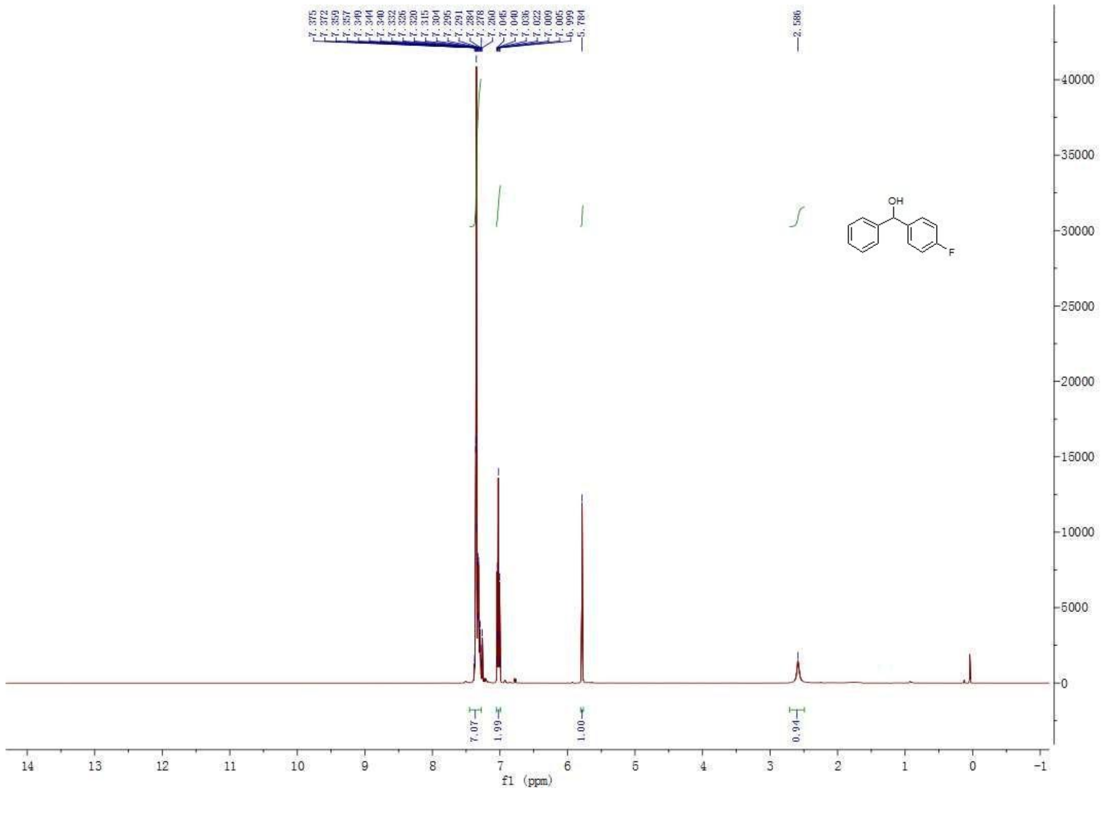

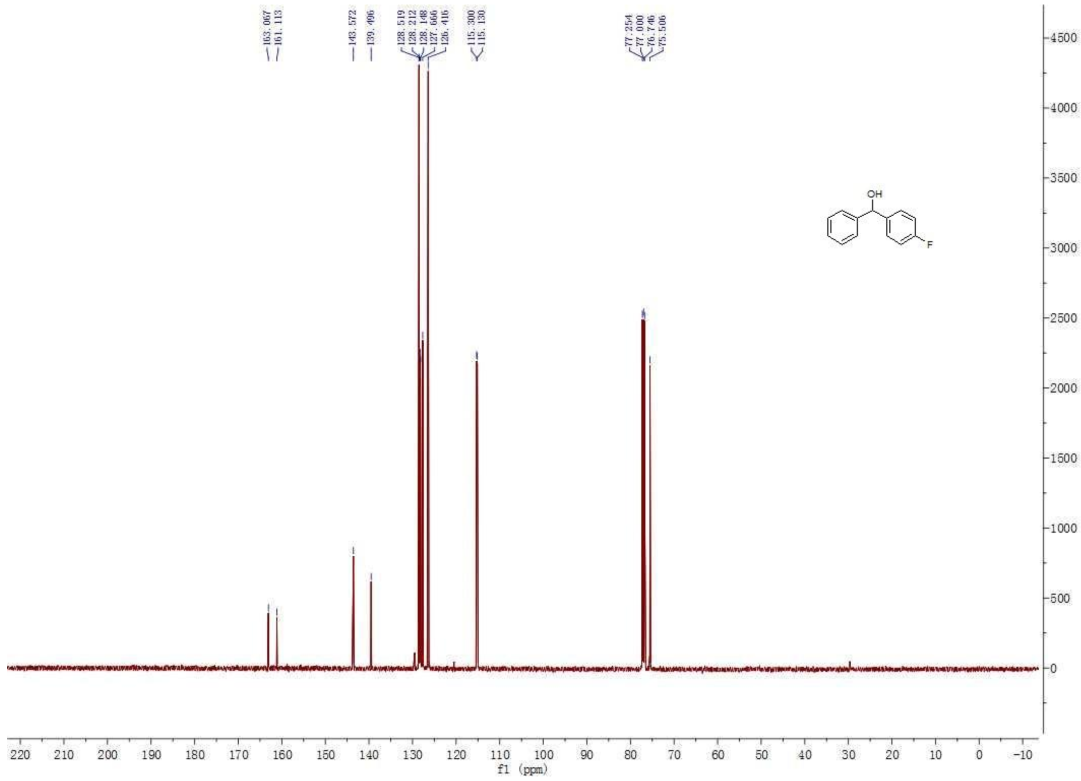

Figure S9. ^{1}H NMR of **3i** (500 MHz, $CDCl_3$) and ^{13}C NMR of **3i** (125 MHz, $CDCl_3$).

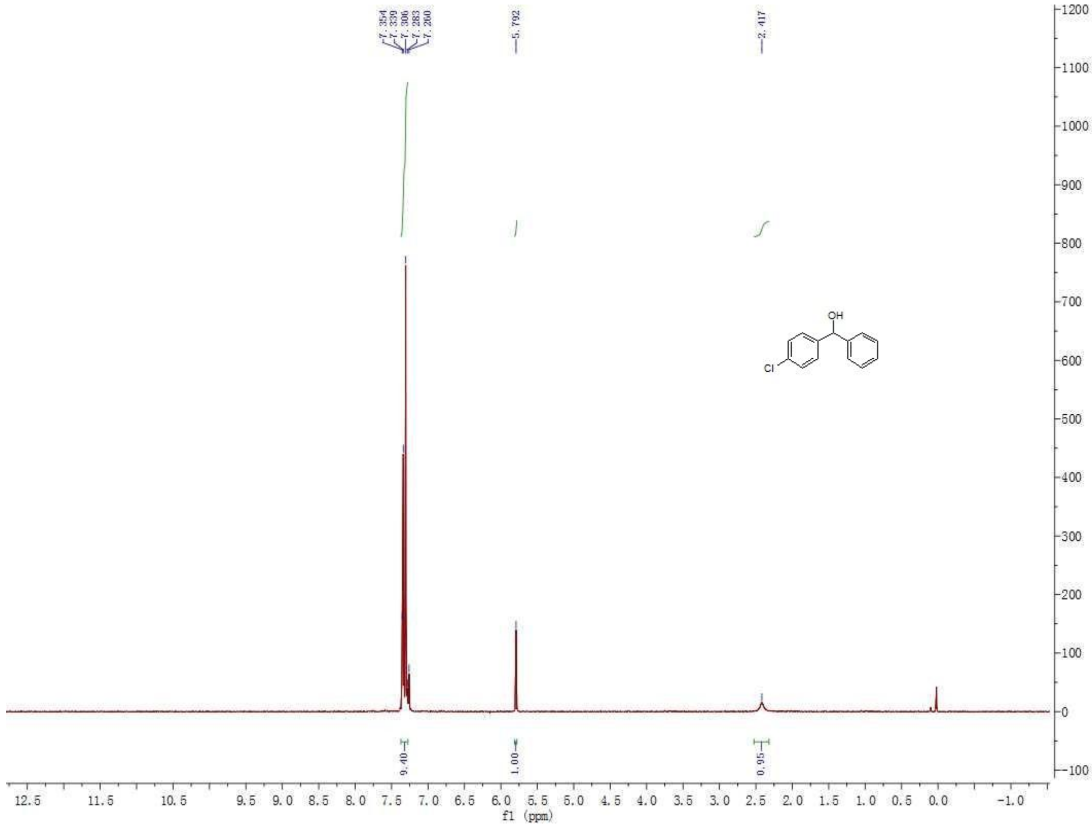

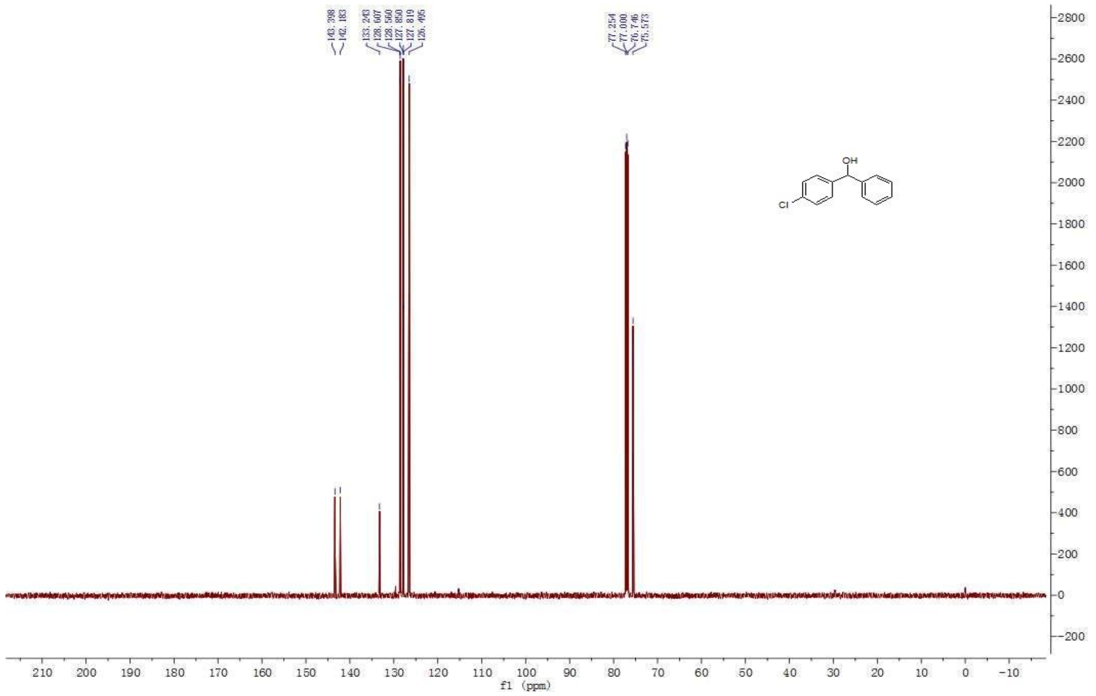

Figure S10. ^{1}H NMR of **3j** (300 MHz, $CDCl_3$) and ^{13}C NMR of **3j** (125 MHz, $CDCl_3$).

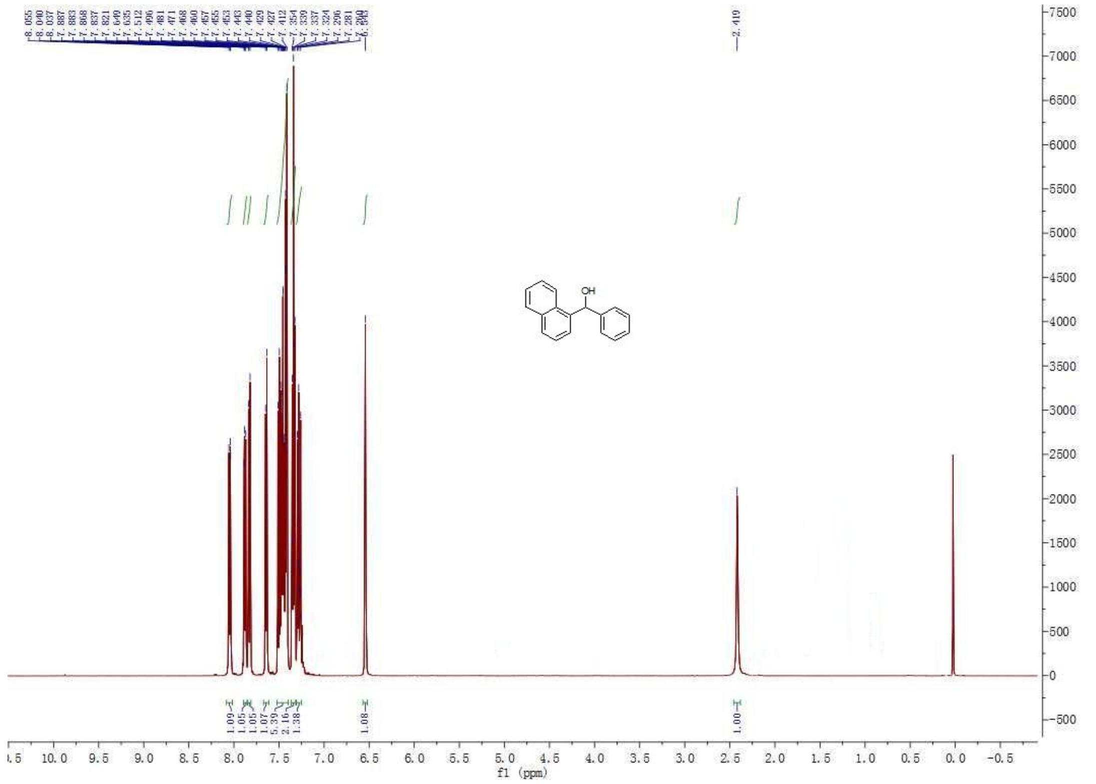

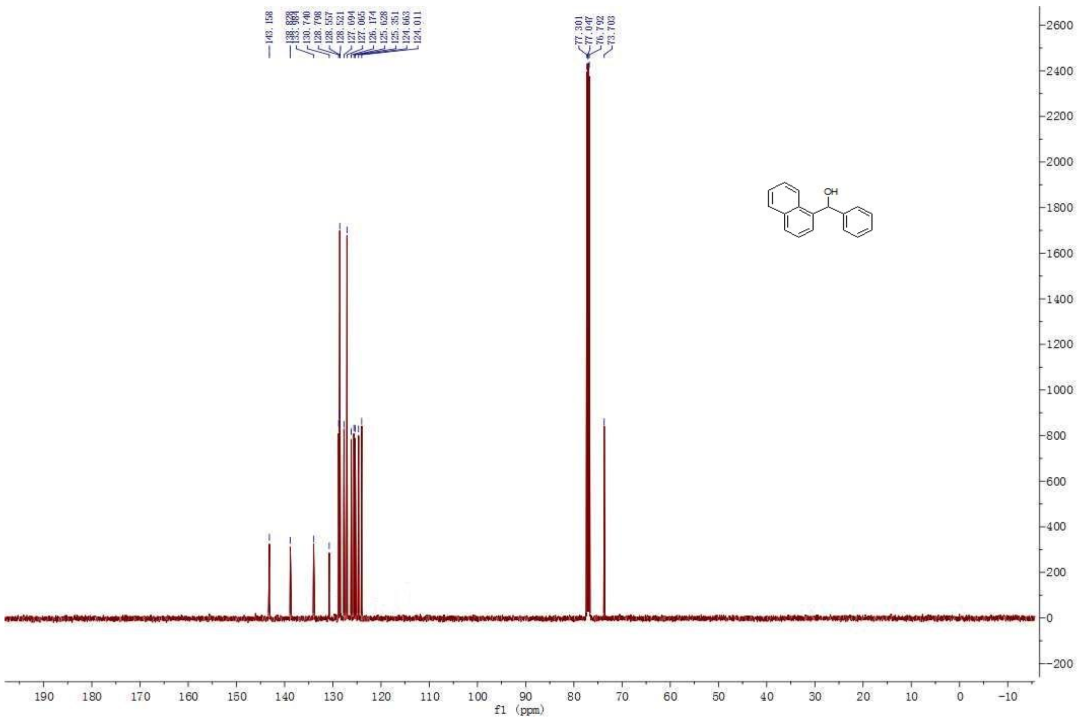

Figure S11. ^{1}H NMR of **3k** (500 MHz, $CDCl_3$) and ^{13}C NMR of **3k** (125 MHz, $CDCl_3$).

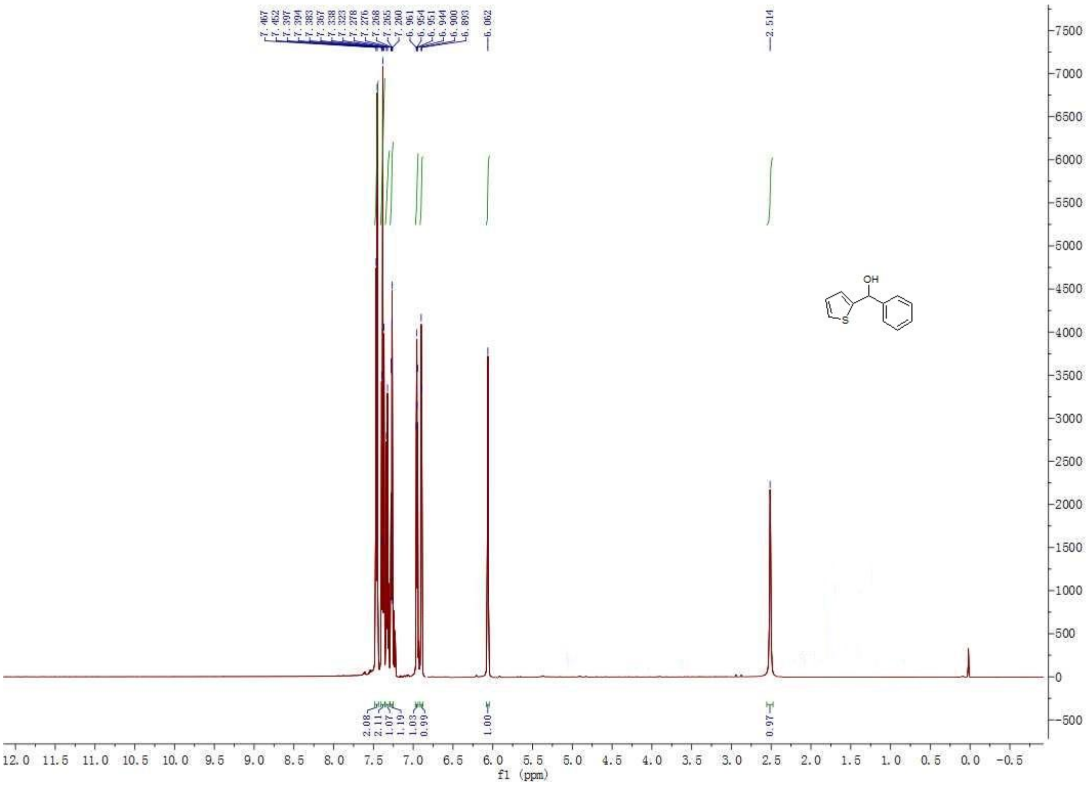

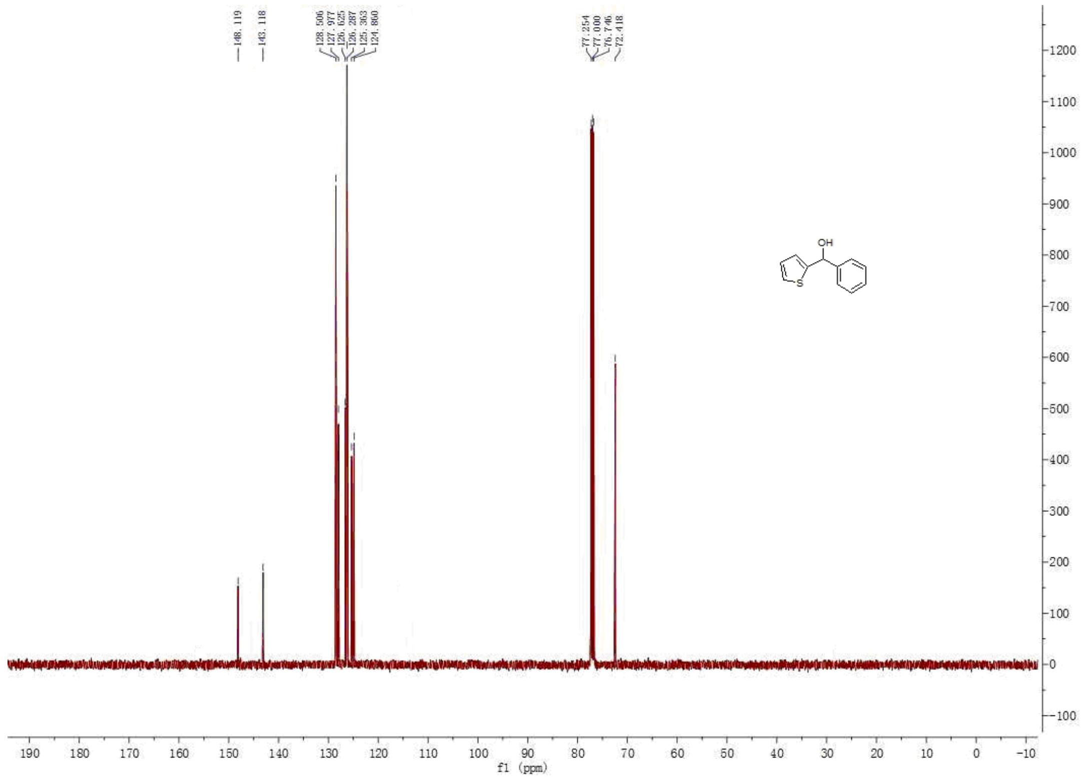

Figure S12. ^{1}H NMR of **3l** (500 MHz, $CDCl_3$) and ^{13}C NMR of **3l** (125 MHz, $CDCl_3$).

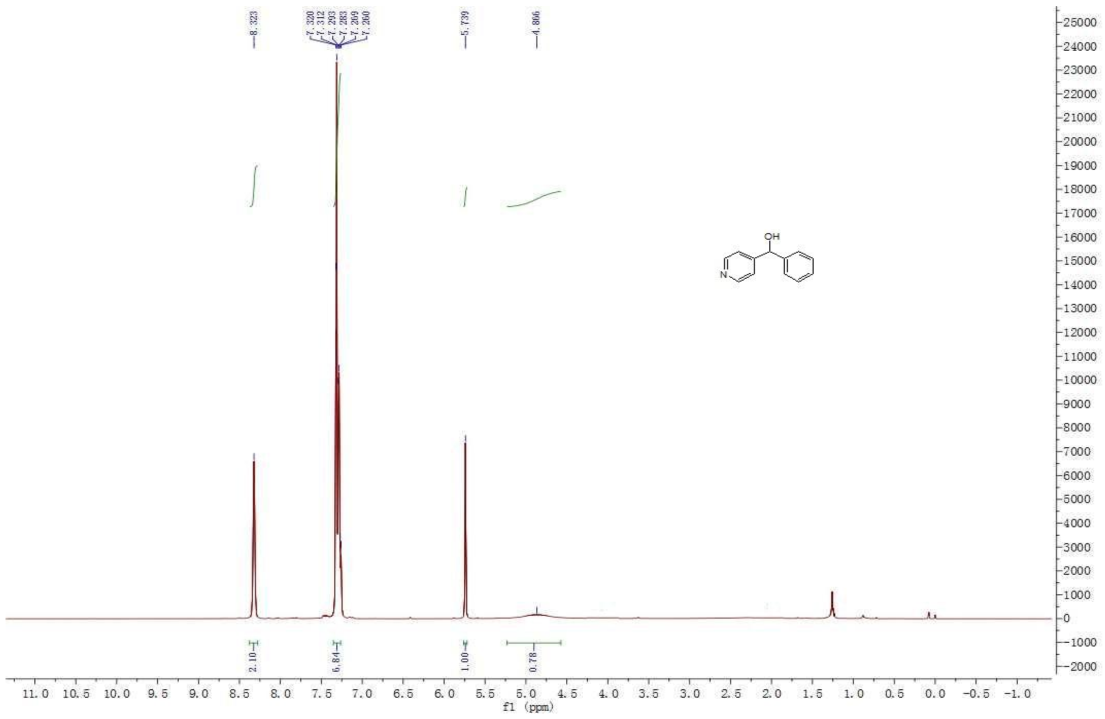

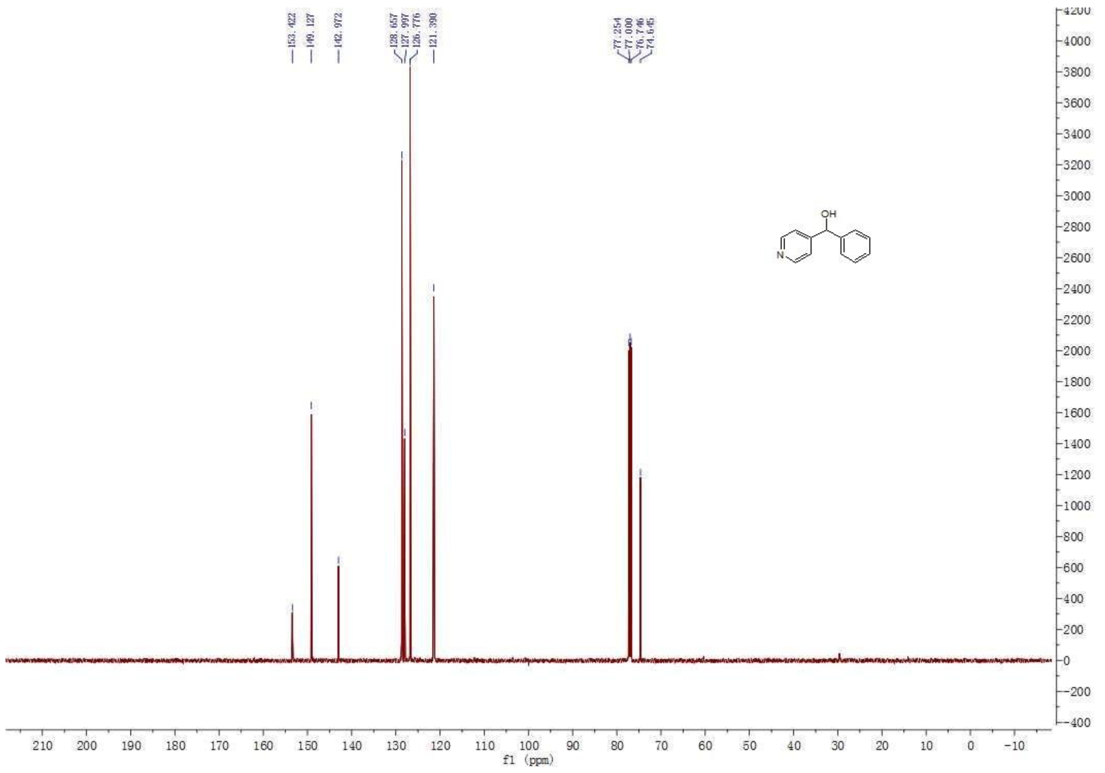

Figure S13. ^{1}H NMR of **3m** (500 MHz, $CDCl_3$) and ^{13}C NMR of **3m** (125 MHz, $CDCl_3$).

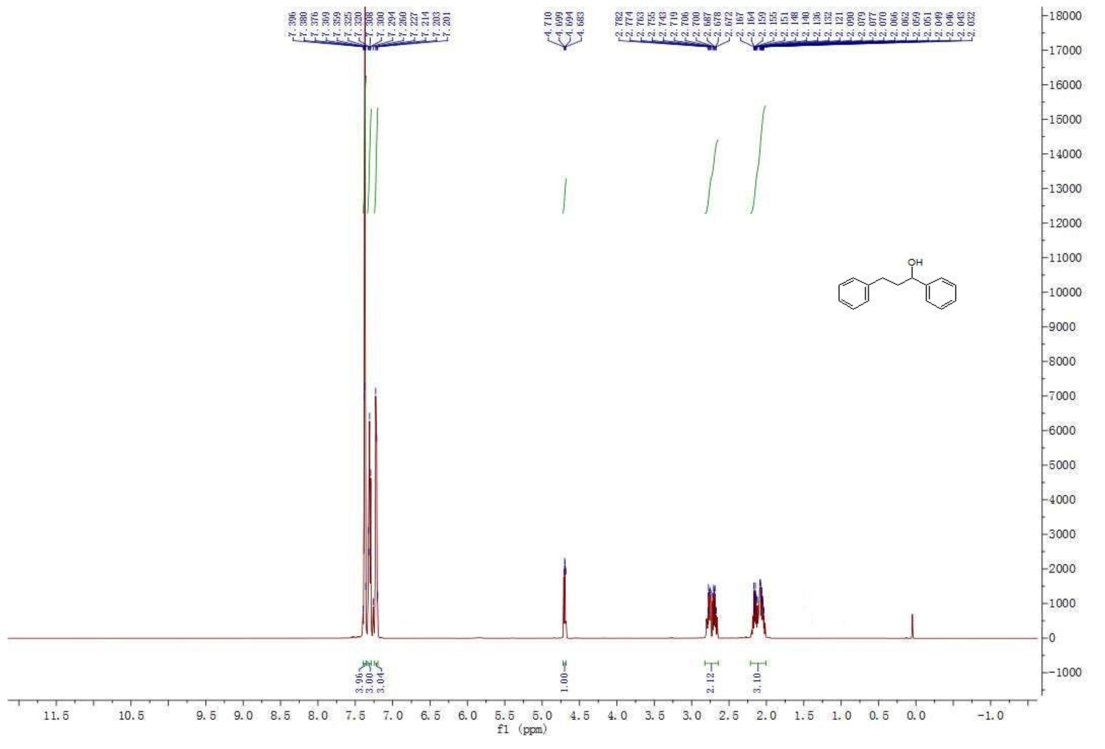

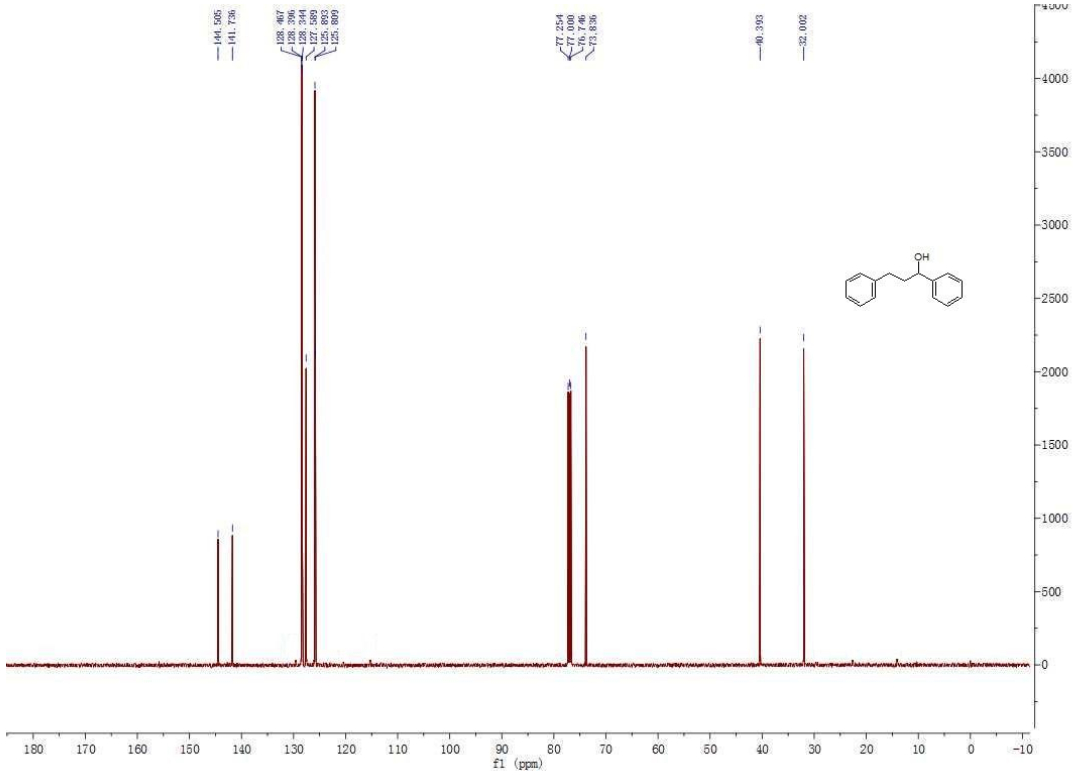

Figure S14. ^{1}H NMR of **3n** (500 MHz, $CDCl_3$) and ^{13}C NMR of **3n** (125 MHz, $CDCl_3$).

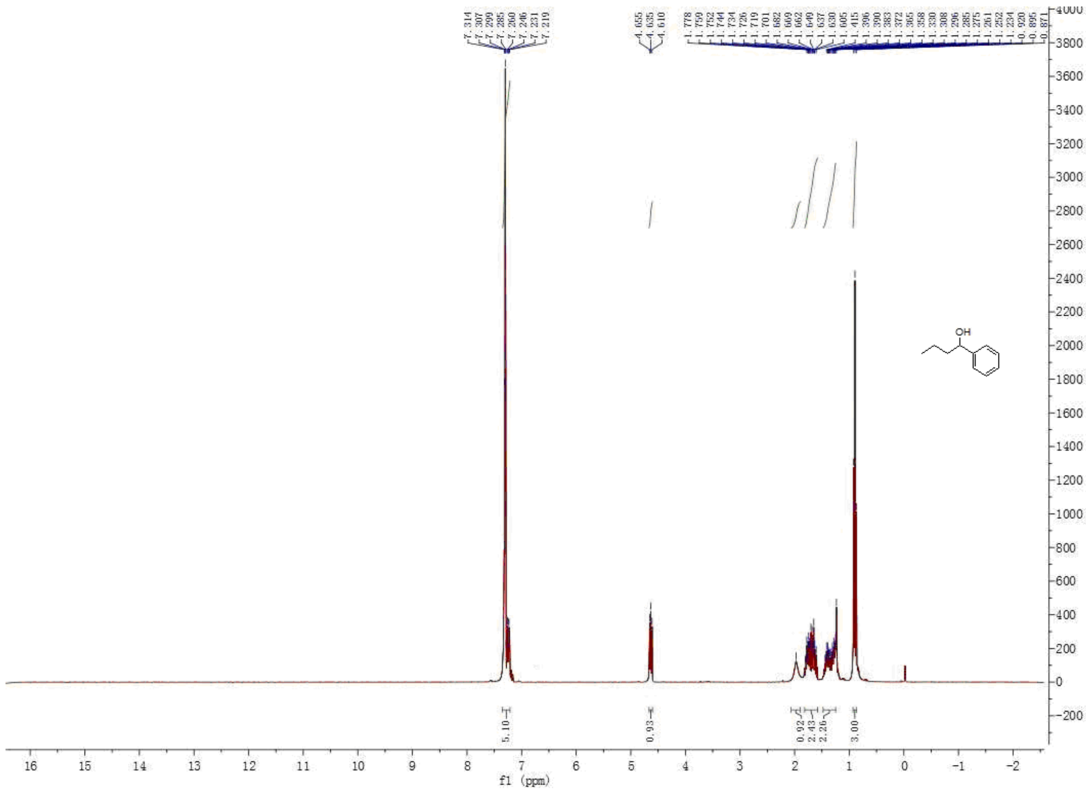

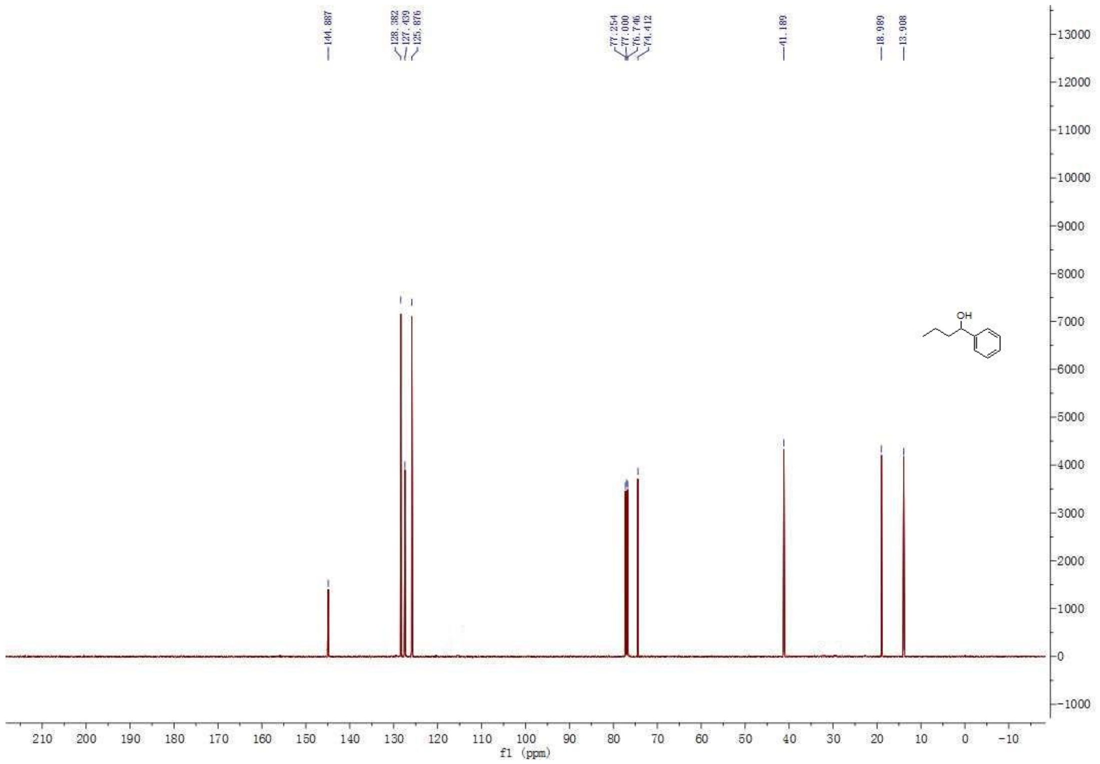

Figure S15. ^{1}H NMR of **3o** (300 MHz, $CDCl_3$) and ^{13}C NMR of **3o** (125 MHz, $CDCl_3$).

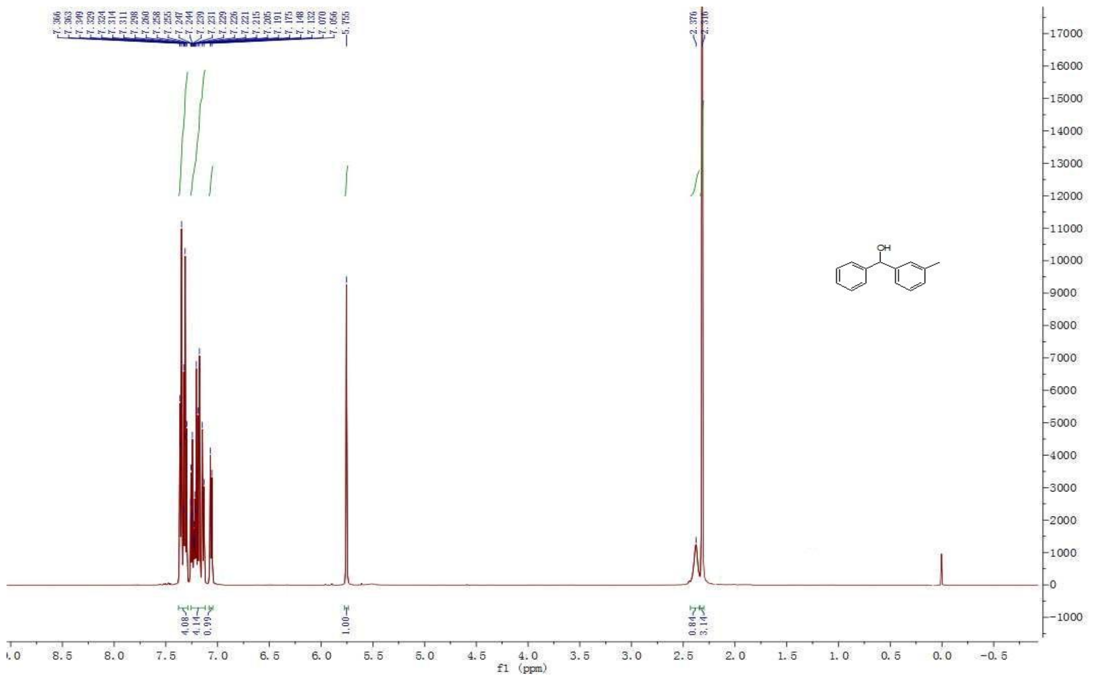

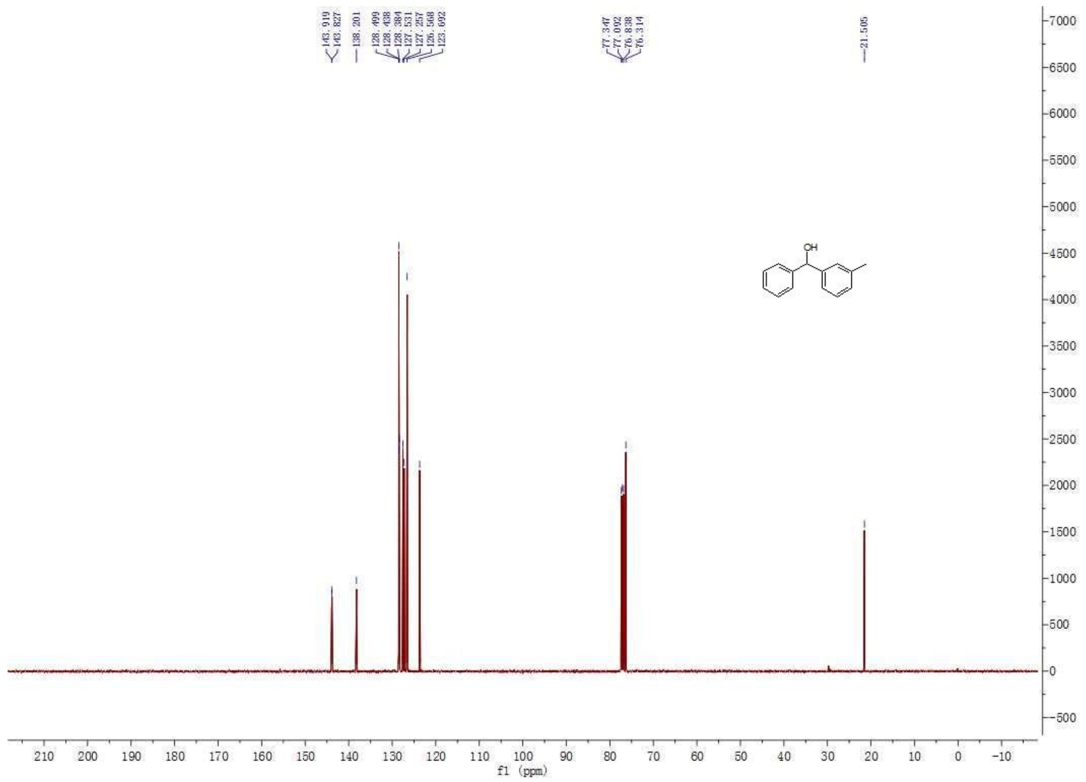

Figure S16. ^{1}H NMR of **3p** (500 MHz, $CDCl_3$) and ^{13}C NMR of **3p** (125 MHz, $CDCl_3$).

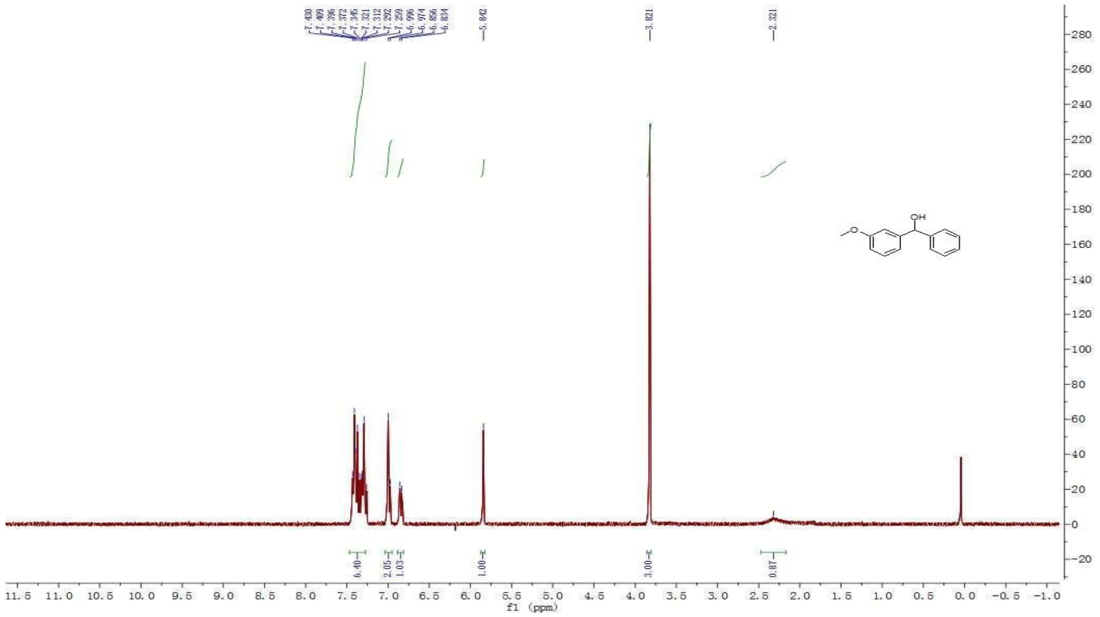

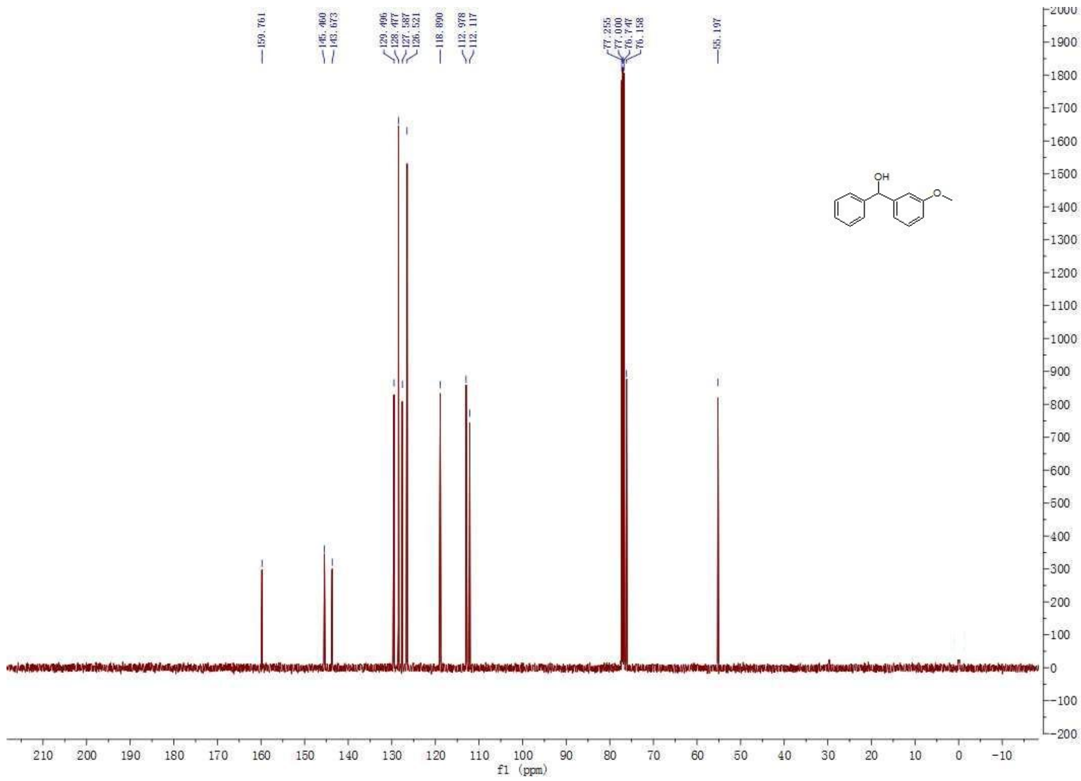

Figure S17. ^{1}H NMR of **3q** (300 MHz, $CDCl_3$) and ^{13}C NMR of **3q** (125 MHz, $CDCl_3$).

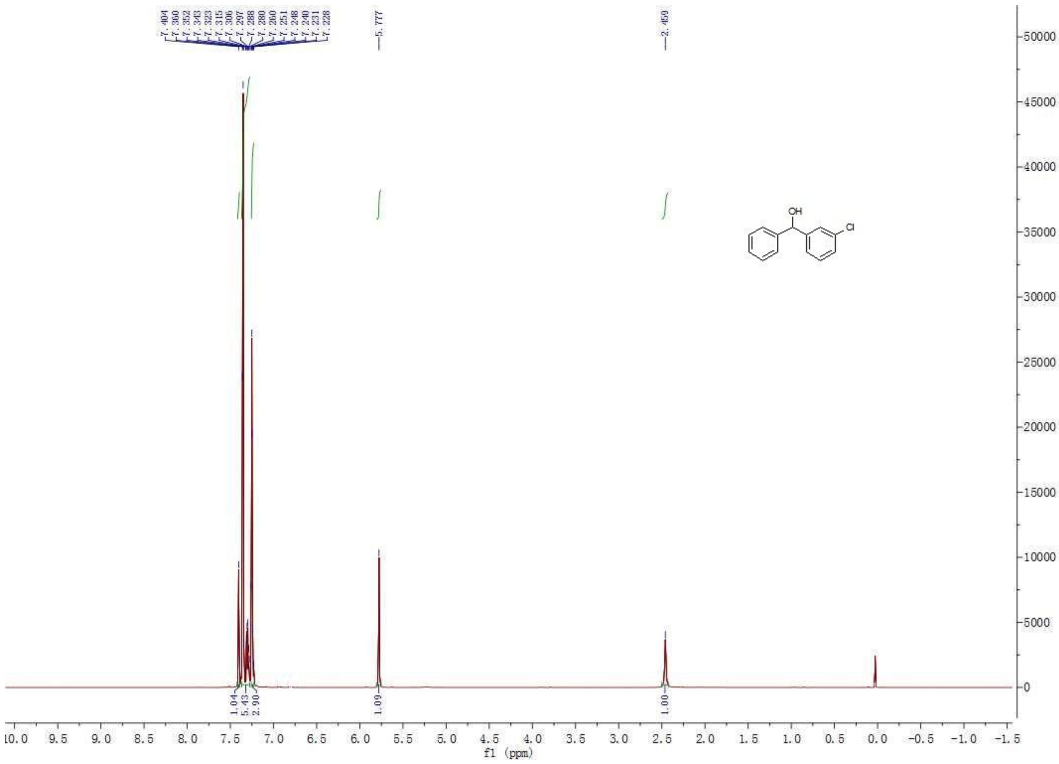

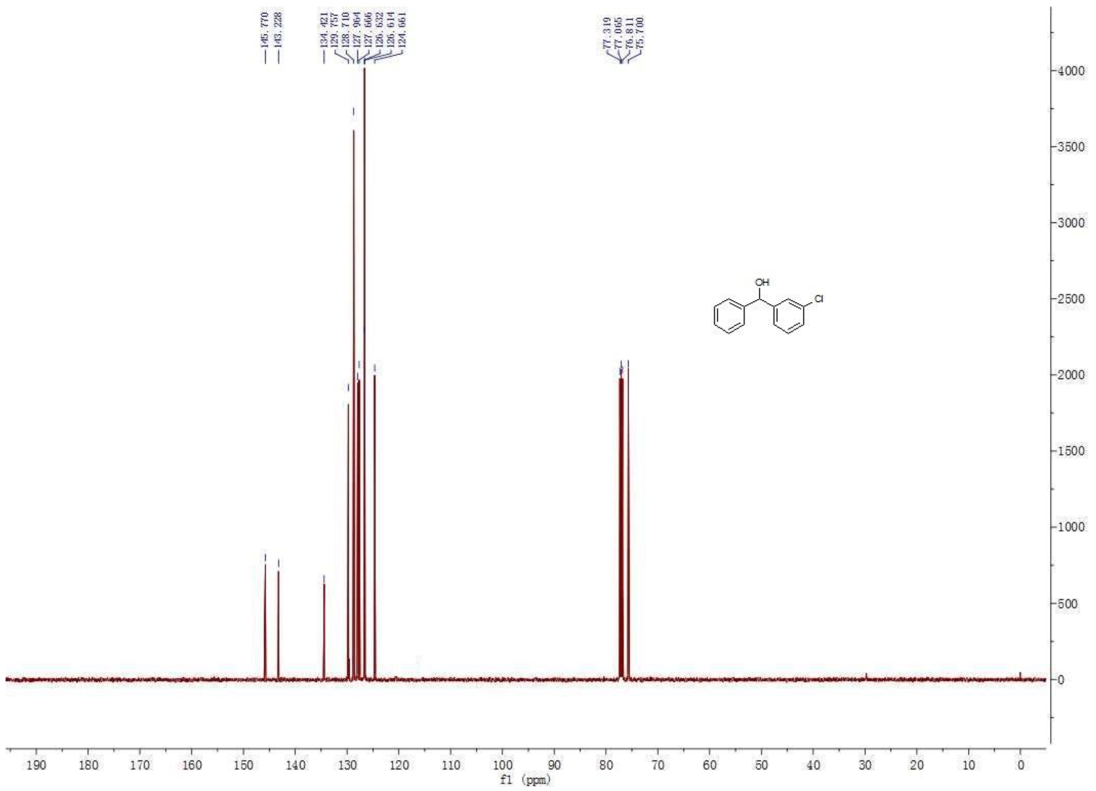

Figure S18. ^{1}H NMR of **3r** (500 MHz, $CDCl_3$) and ^{13}C NMR of **3r** (125 MHz, $CDCl_3$).

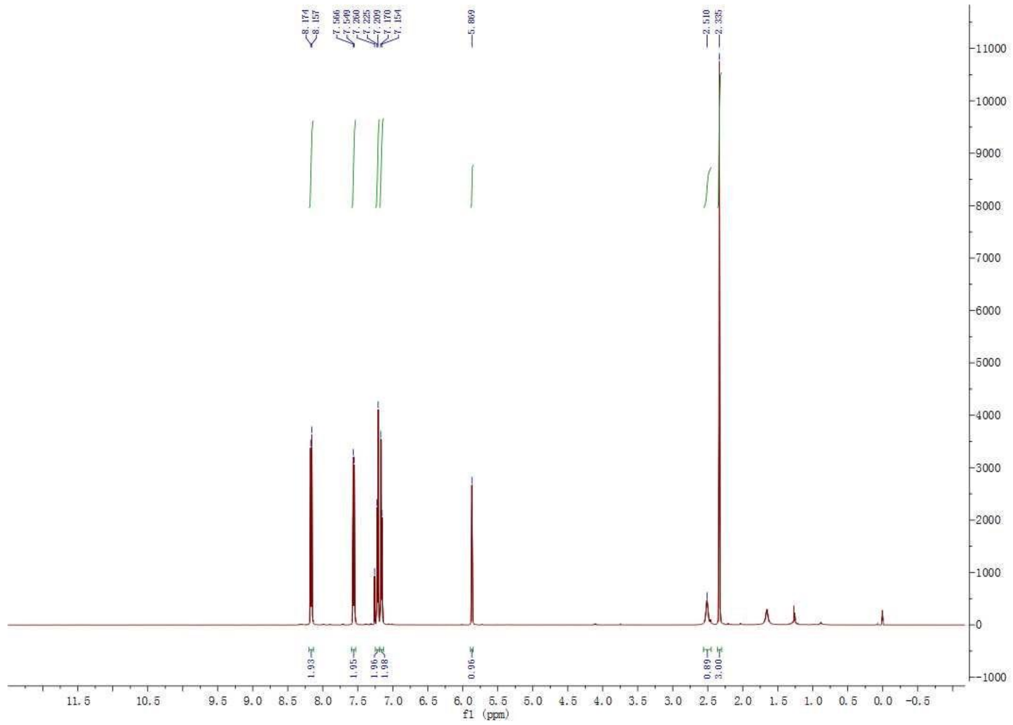

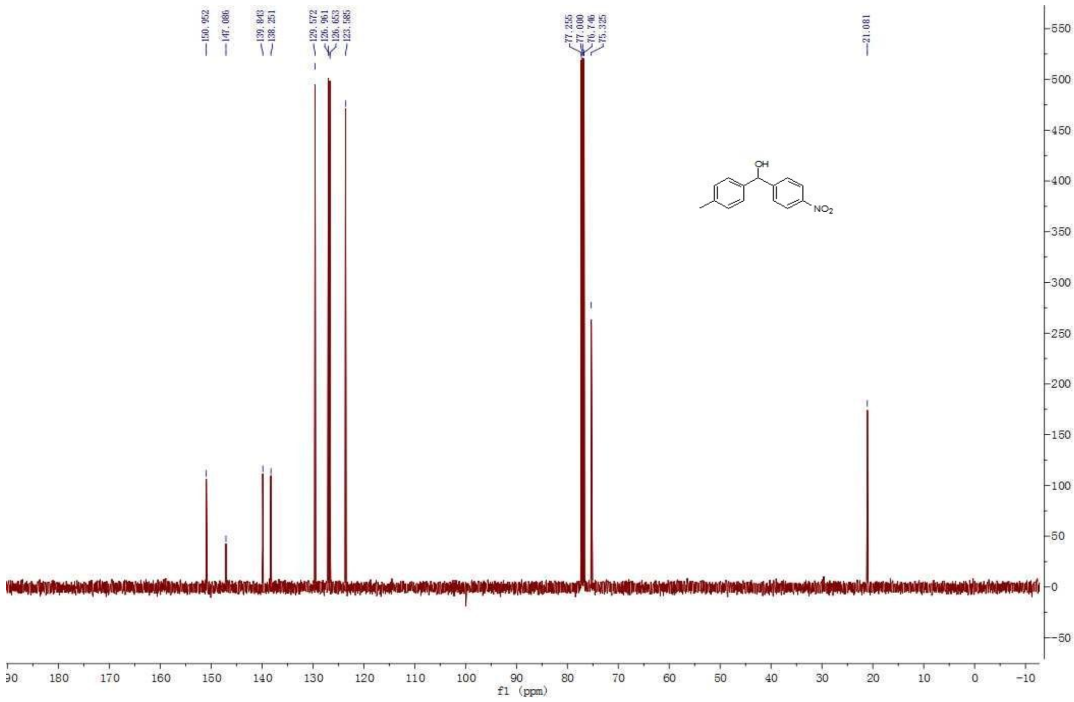

Figure S19. ^{1}H NMR of **3s** (500 MHz, $CDCl_3$) and ^{13}C NMR of **3s** (125 MHz, $CDCl_3$).

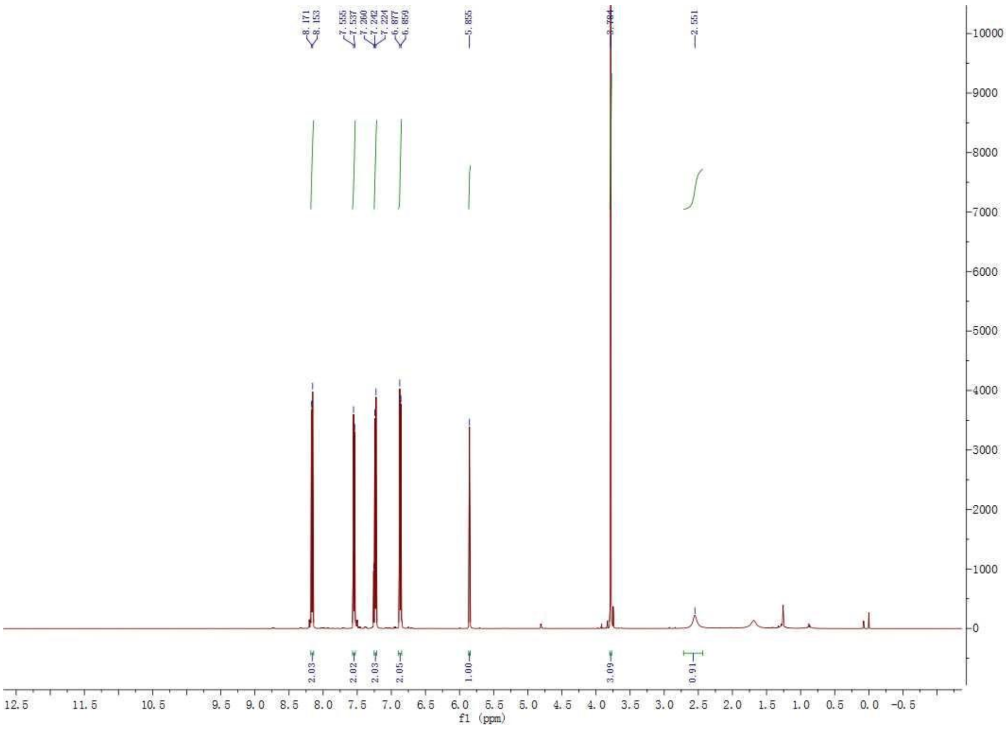

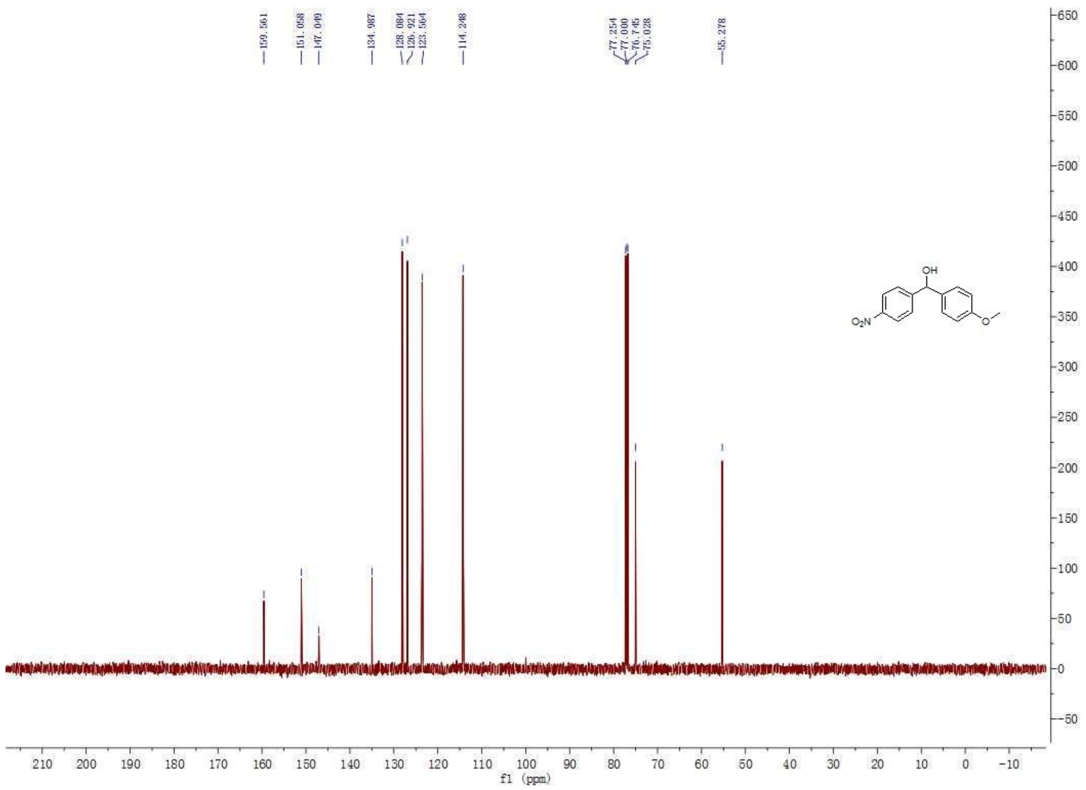

Figure S20. ^{1}H NMR of **3t** (500 MHz, $CDCl_3$) and ^{13}C NMR of **3t** (125 MHz, $CDCl_3$).

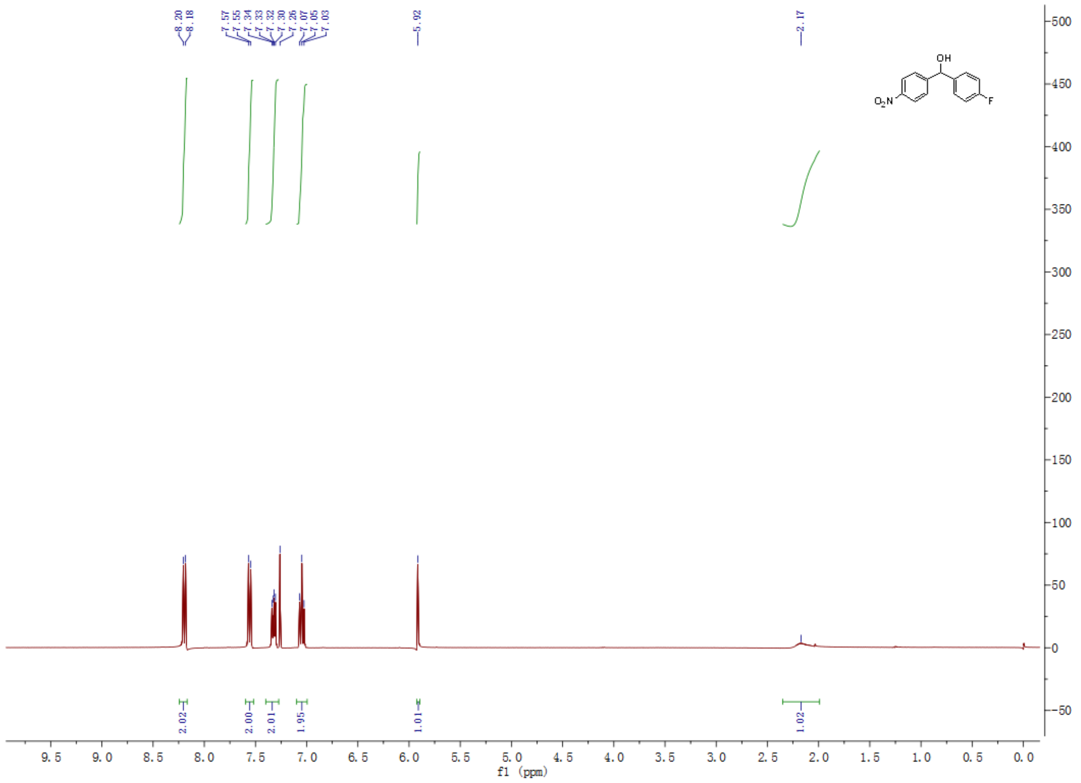

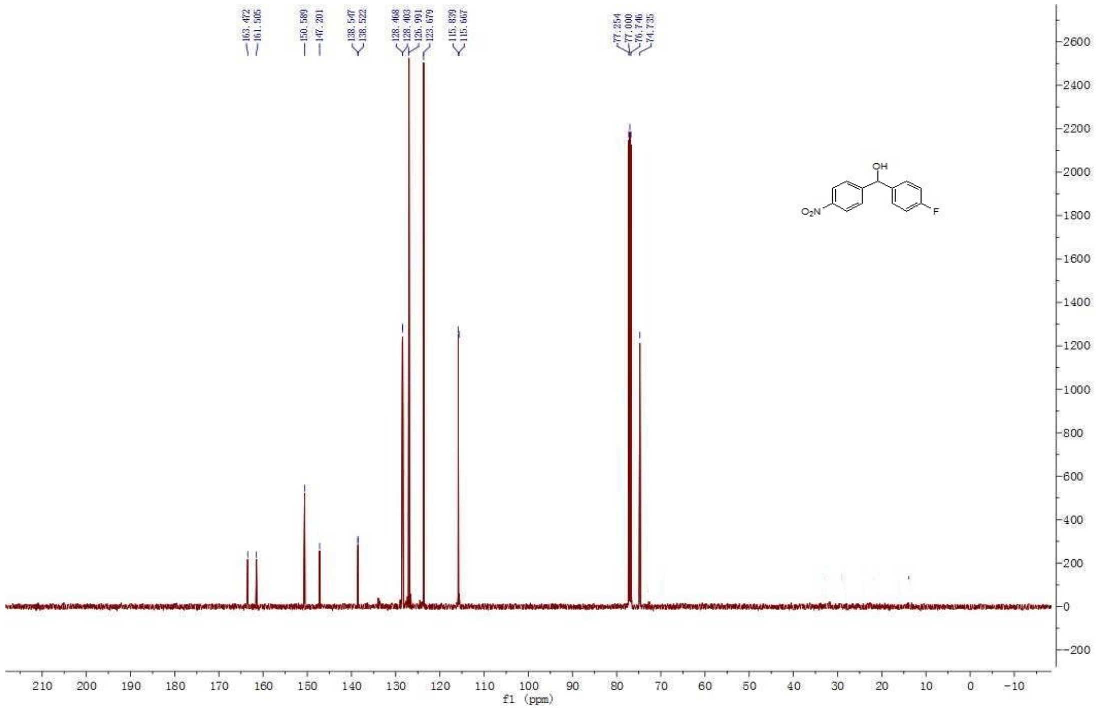

Figure S21. ^{1}H NMR of **3u** (500 MHz, $CDCl_3$) and ^{13}C NMR of **3u** (125 MHz, $CDCl_3$).

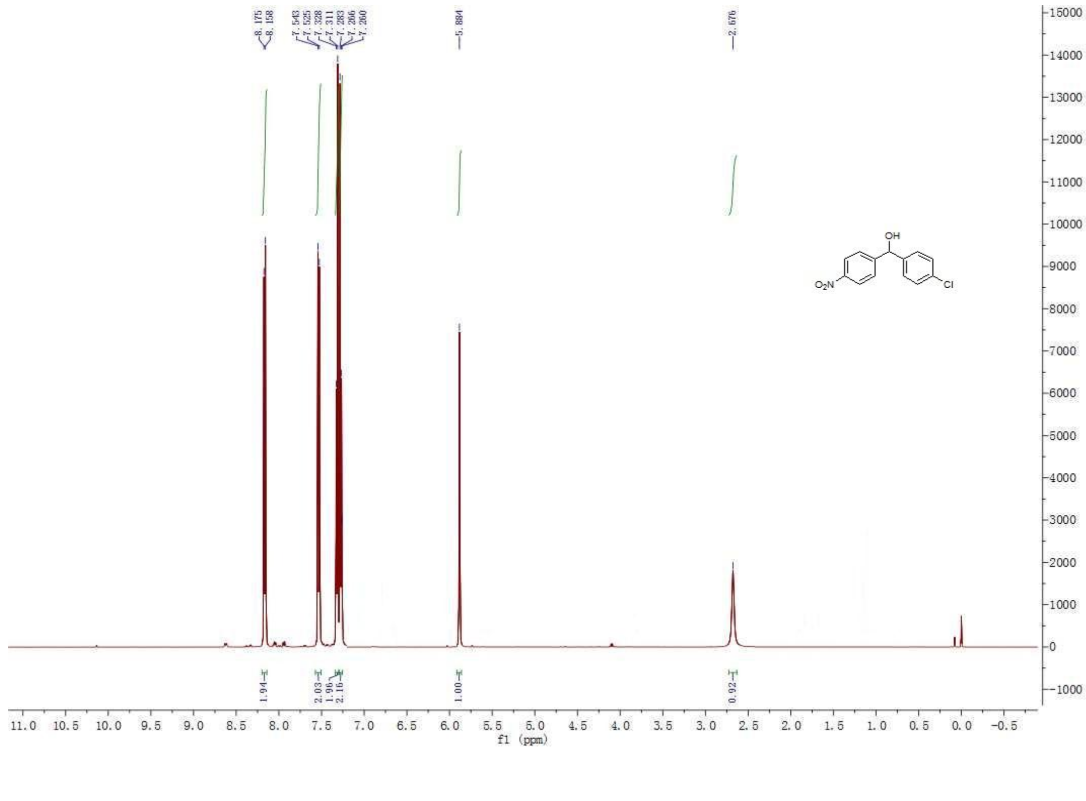

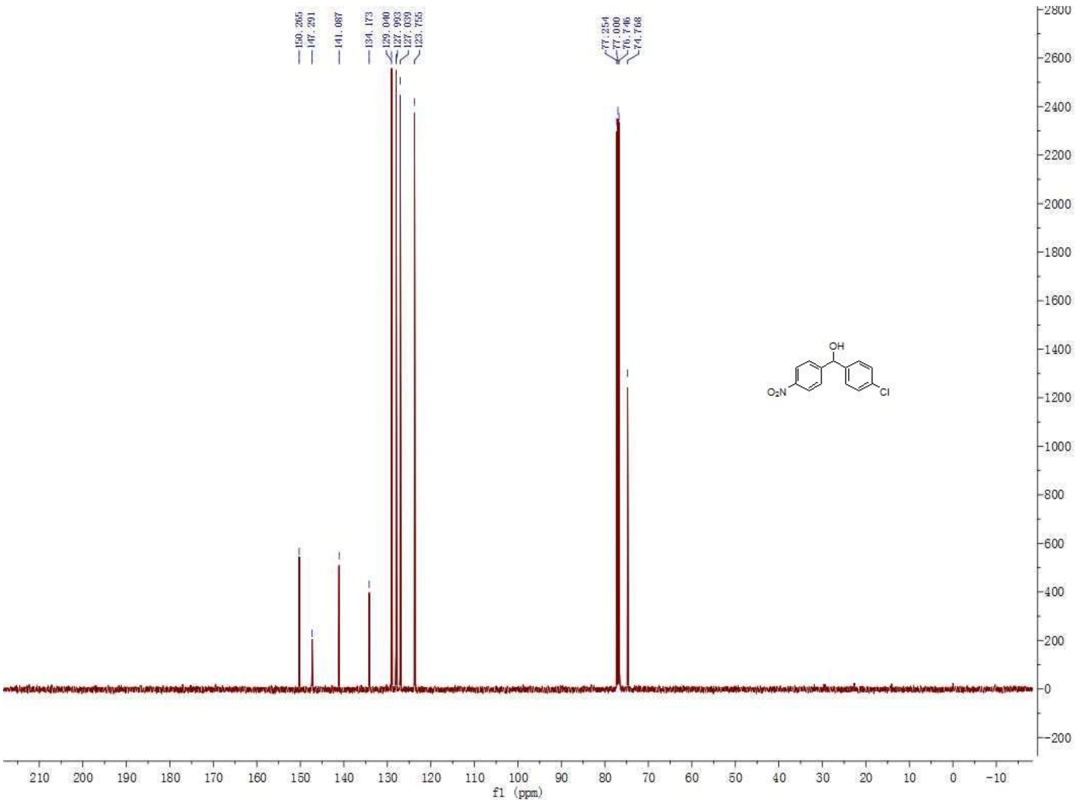

Figure S22. ^{1}H NMR of **3v** (500 MHz, $CDCl_3$) and ^{13}C NMR of **3v** (125 MHz, $CDCl_3$).

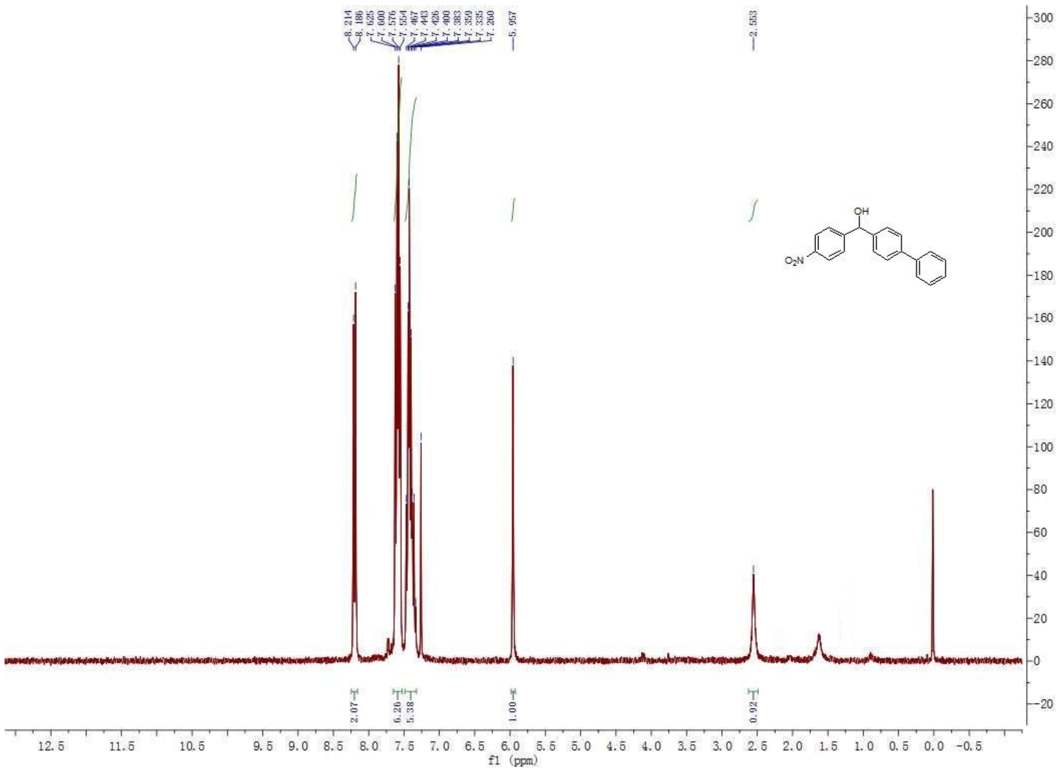

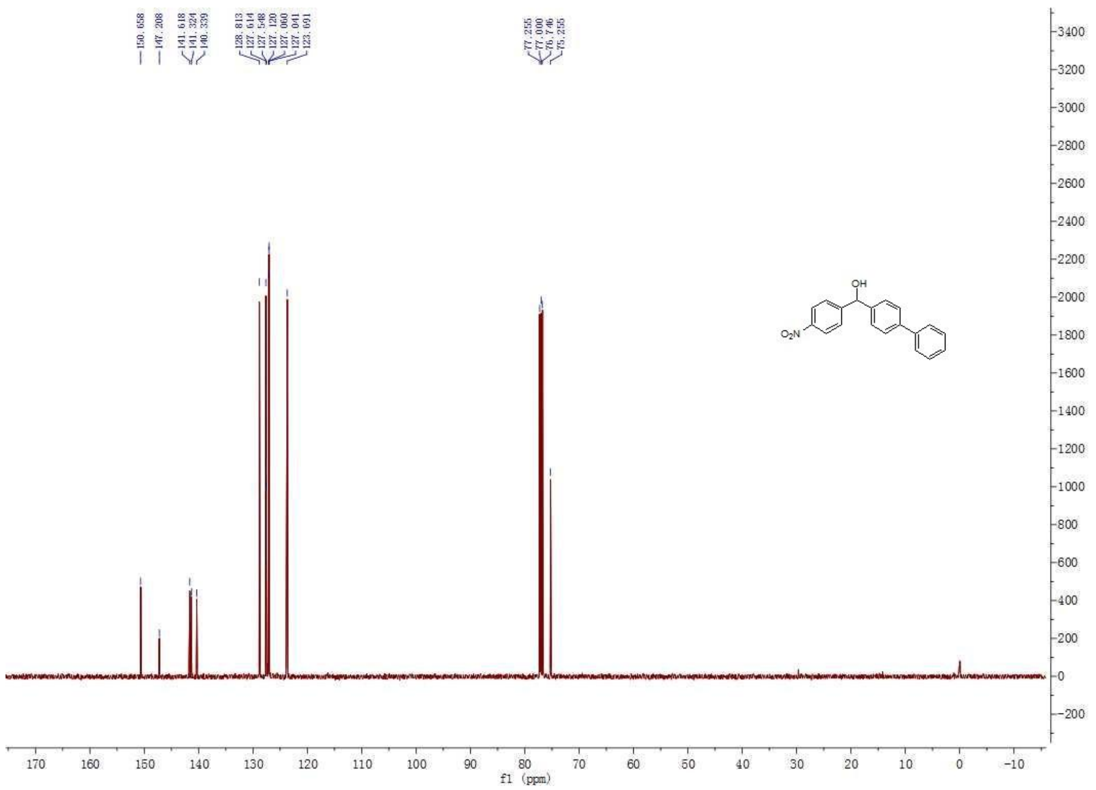

Figure S23. ^{1}H NMR of **3w** (300 MHz, $CDCl_3$) and ^{13}C NMR of **3w** (125 MHz, $CDCl_3$).

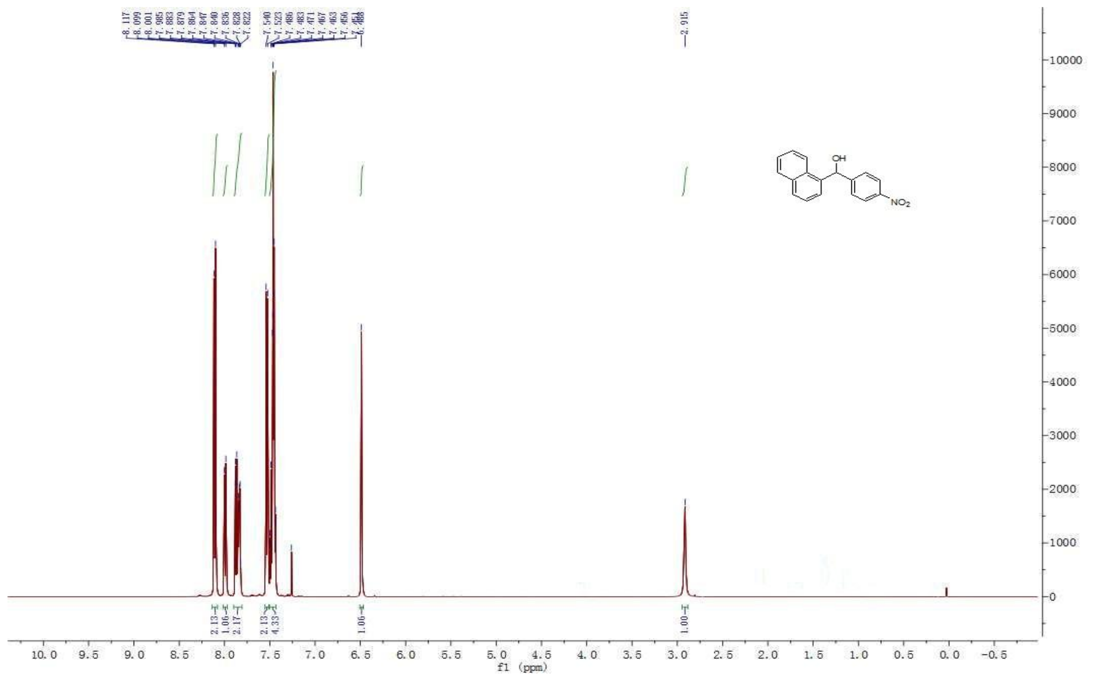

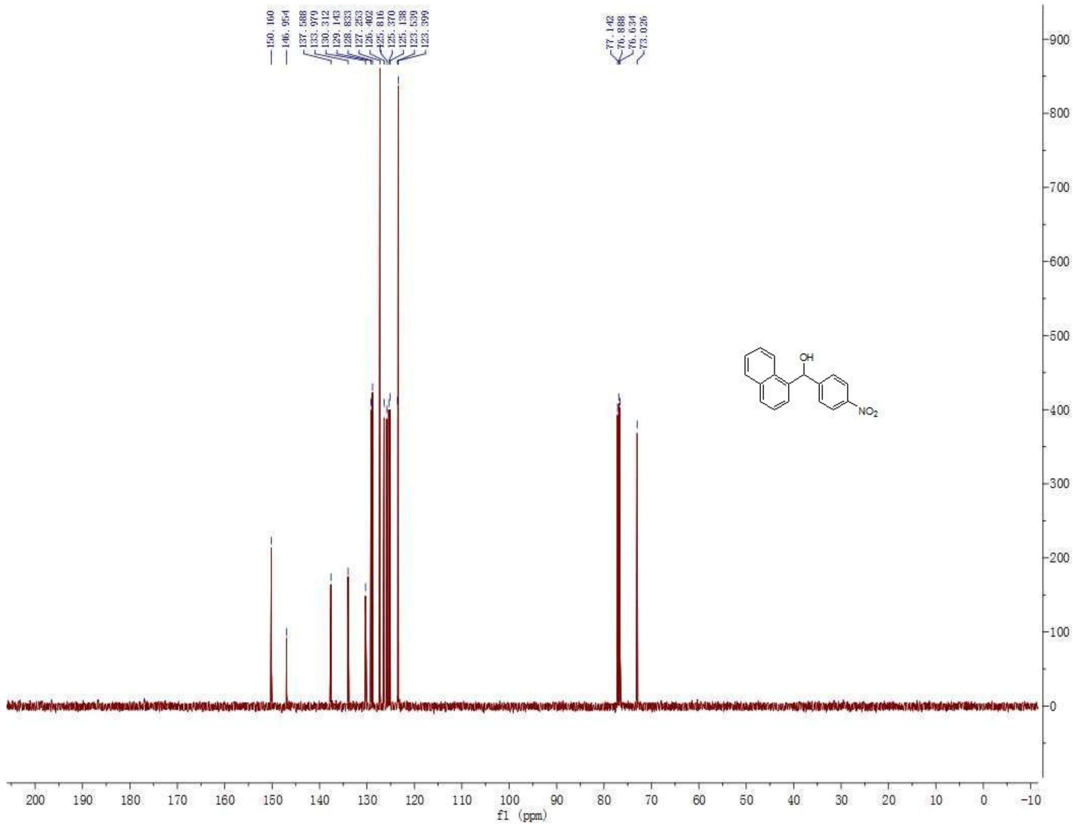

Figure S24. ^{1}H NMR of **3x** (500 MHz, $CDCl_3$) and ^{13}C NMR of **3x** (125 MHz, $CDCl_3$).

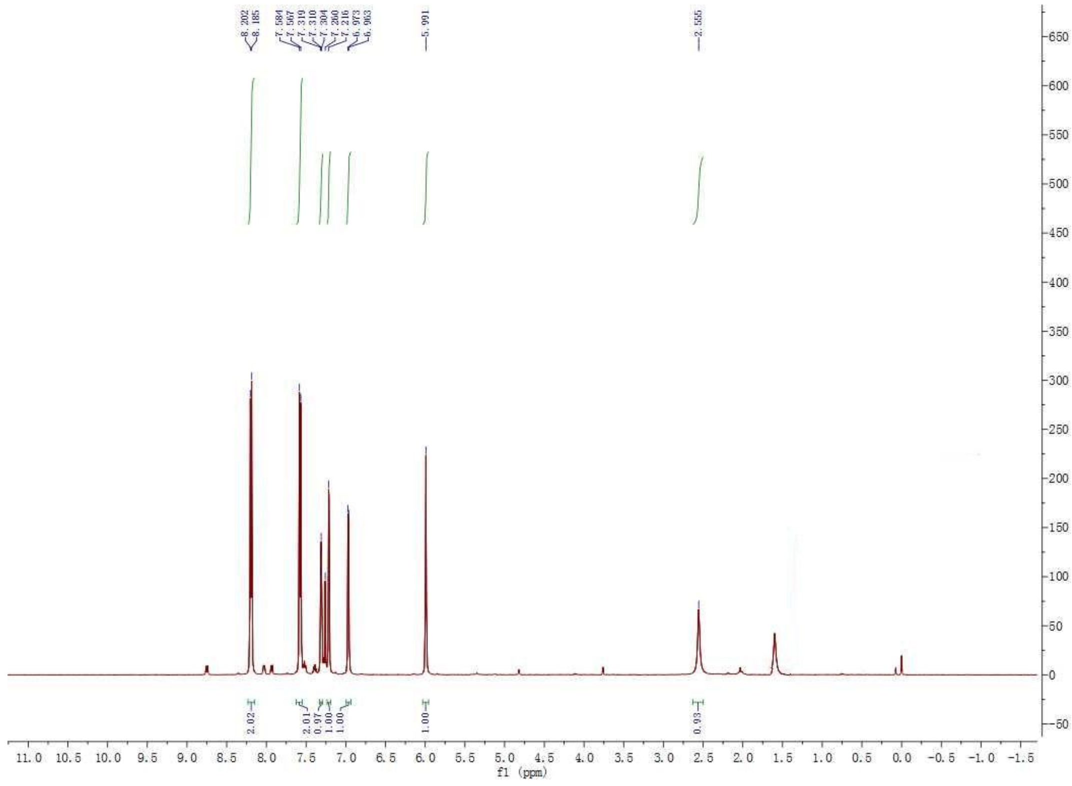

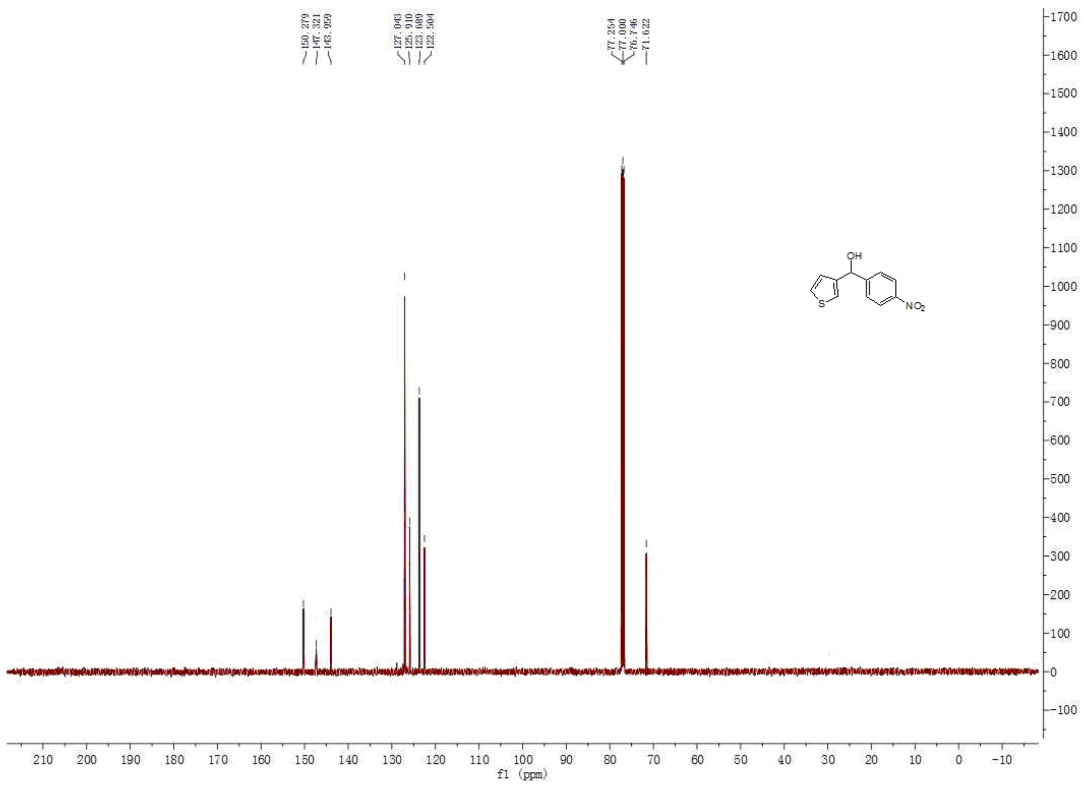

Figure S25. ^{1}H NMR of **3y** (500 MHz, $CDCl_3$) and ^{13}C NMR of **3y** (125 MHz, $CDCl_3$).
